# Exosomal mir-126-3p derived from endothelial cells induces ion channel dysfunction by targeting RGS3 signaling in cardiomyocytes: a novel mechanism in Takotsubo cardiomyopathy

**DOI:** 10.1186/s13287-025-04157-0

**Published:** 2025-02-04

**Authors:** Xuehui Fan, Guoqiang Yang, Yinuo Wang, Haojie Shi, Katja Nitschke, Katherine Sattler, Mohammad Abumayyaleh, Lukas Cyganek, Philipp Nuhn, Thomas Worst, Bin Liao, Gergana Dobreva, Daniel Duerschmied, Xiaobo Zhou, Ibrahim El-Battrawy, Ibrahim Akin

**Affiliations:** 1 Department of Cardiology, Angiology, Hemostaseology and Medical Intensive Care, Medical Faculty Mannheim, University Medical Centre Mannheim (UMM), Heidelberg University, Mannheim, Germany; 2 Key Laboratory of Medical Electrophysiology of the Ministry of Education, Medical Electrophysiological Key Laboratory of Sichuan Province, Institute of Cardiovascular Research, Southwest Medical University, Luzhou, Sichuan China; 3 Department of Cardiology, The Affiliated Hospital of Southwest Medical University, Luzhou, 646000 China; 4 European Center for AngioScience (ECAS) and German Center for Cardiovascular Research (DZHK) Partner Site Heidelberg/Mannheim, Mannheim, Germany; 5 Acupuncture and Rehabilitation Department, The Affiliated Traditional Chinese Medicine Hospital of Southwest Medical University, Luzhou, 646000 China; 6 Department of Cardiovascular Genomics and Epigenomics, European Center for Angioscience (ECAS), Medical Faculty Mannheim, Heidelberg University, Mannheim, Germany; 7 Department of Urology and Urosurgery, Medical Faculty Mannheim, Heidelberg University, Mannheim, Germany; 8 Stem Cell Unit, Clinic for Cardiology and Pneumology, University Medical Center Göttingen, Göttingen, Germany; 9 DZHK (German Center for Cardiovascular Research), Partner Site, Göttingen, Germany; 10 Department of Cardiac Macrovascular Surgery, Affiliated Hospital of Southwest Medical University, Sichuan, 646000 China; 11 Department of Cardiology and Angiology, Bergmannsheil University Hospitals, Ruhr University of Bochum, 44789 Bochum, Germany; 12 Institute of Physiology, Department of Cellular and Translational Physiology, Medical Faculty and Institut für Forschung und Lehre (IFL), Molecular and Experimental Cardiology, Ruhr University Bochum, Bochum, Germany

**Keywords:** Takotsubo cardiomyopathy, Catecholamine excess, Exosomes, miRNA-126-3p, Human-induced pluripotent stem cell-derived cardiomyocytes

## Abstract

**Background:**

Takotsubo cardiomyopathy (TTC) is marked by an acute, transient, and reversible left ventricular systolic dysfunction triggered by stress, with endothelial dysfunction being one of its pathophysiological mechanisms. However, the precise molecular mechanism underlying the interaction between endothelial cells and cardiomyocytes during TTC remains unclear. This study reveals that exosomal miRNAs derived from endothelial cells exposed to catecholamine contribute to ion channel dysfunction in the setting of TTC.

**Methods:**

Human-induced pluripotent stem cell-derived cardiomyocytes (hiPSC-CMs) were treated with epinephrine (Epi) or exosomes (Exo) from Epi-treated human cardiac microvascular endothelial cells (HCMECs) or Exo derived from HCMECs transfected with miR-126-3p. The immunofluorescence staining, flow cytometry, qPCR, single-cell contraction, intracellular calcium transients, patch-clamp, dual luciferase reporter assay and western blot were performed for the study.

**Results:**

Modeling TTC with high doses of epinephrine (Epi) treatment in hiPSC-CMs shows suppression of depolarization velocity (Vmax), prolongation of action potential duration (APD), and induction of arrhythmic events. Exo derived from HCMECs treated with Epi (Epi-exo) mimicked or enhanced the effects of Epi. Epi exposure led to elevated levels of miR-126-3p in both HCMECs and their exosomes. Exo enriched with miR-126-3p demonstrated similar effects as Epi-exo, establishing the crucial role of miR-126-3p in the mechanism of Epi-exo. Dual luciferase reporter assay coupled with gene mutation techniques identified that miR-126-3p was found to target the regulator of G-protein signaling 3 (RGS3) gene. Western blot and qPCR analyses confirmed that miR-126-3p-mimic reduced RGS3 expression in both HCMECs and hiPSC-CMs, indicating miR-126-3p inhibits RGS3 signaling. Additionally, miR-126-3p levels were significantly higher in the serum of TTC patients compared to healthy controls and patients who had recovered from TTC.

**Conclusions:**

Our study is the first to reveal that exosomal miR-126-3p, originating from endothelial cells, contributes to ion channel dysfunction by regulating RGS3 signaling in cardiomyocytes. These findings provide new perspectives on the pathogenesis of TTC and suggest potential therapeutic targets for treatment.

**Graphical Abstract:**

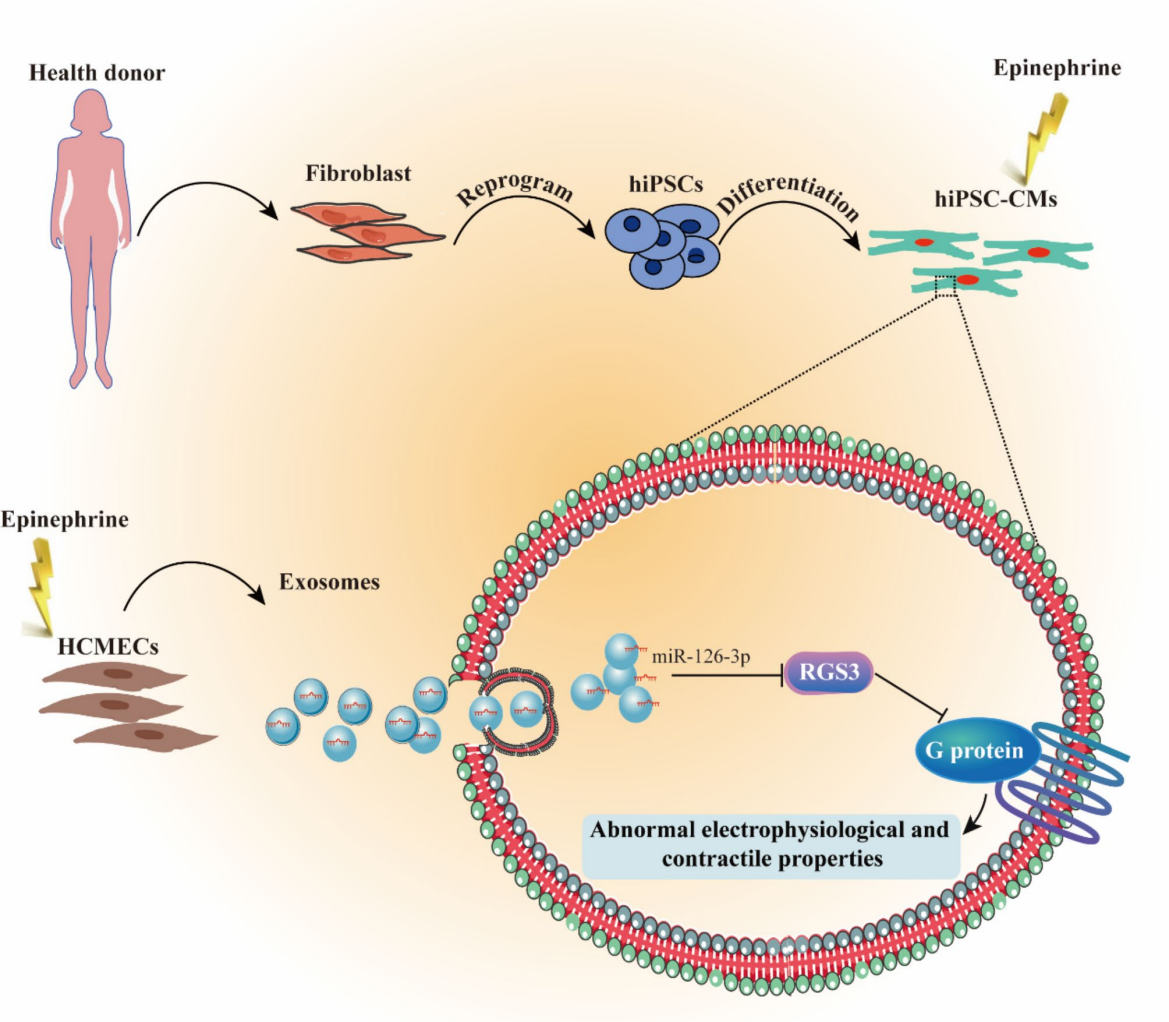

**Supplementary Information:**

The online version contains supplementary material available at 10.1186/s13287-025-04157-0.

## Introduction

Takotsubo cardiomyopathy (TTC) is an acute cardiac disorder marked by a transient and reversible left ventricular systolic dysfunction. It commonly presents with apical ballooning and abnormal electrocardiograms (ECG), such as ST-segment elevation or T-wave inversion, without evidence of obstructive coronary artery disease, and is often triggered by emotional or physical stress [1, 2]. The disorder is characterized by hypercontractility in the basal and mid-ventricular regions, with wall motion abnormalities in the apical region [3]. Despite similarities in clinical characteristics and disease course between genders, physical stress tends to precipitate TTC with much worse outcomes in male patients compared to female patients [4, 5]. The 30-day mortality rate in TTC patients lies between ST-segment elevation myocardial infarction (STEMI) and non-STEMI (NSTEMI) patients [6]. Therefore, understanding the underlying pathophysiology and molecular mechanisms of TTC is crucial to enhancing its clinical management.

The pathogenesis of TTC is thought to involve a range of mechanisms, including increased levels of circulating plasma catecholamines and their metabolites, coronary microvascular endothelial dysfunction/epicardial coronary vasospasm, sympathetic overactivity, signal trafficking/biased agonism, inflammation, genetic predisposition, basal hypercontractility with left ventricular outflow tract obstruction, estrogen deficiency, and thyroid dysfunction [3, 7–10]. TTC likely arises from the interplay of these various factors, but its exact mechanisms remain incompletely understood. Endothelial cells, a predominant non-myocyte cell type within the heart, can produce and secrete catecholamines [11]. Dysfunction in these endothelial cells, characterized by an imbalance between vasoconstriction and vasodilation factors [12], may play a crucial role in the pathogenesis of TTC. Currently, the connection between endothelial dysfunction and TTC is mostly supported by clinical evidence. Nevertheless, the specific impact of endothelial dysfunction on the myocardium independent of blood supply remains unclear.

Endothelial cells and cardiomyocytes are located in close proximity and engage in communication through various mechanisms, including paracrine signaling, direct cell-to-cell contact, or secretion of molecules or vesicles [12]. Endothelial cell-released exosomes, triggered by high glucose levels, have been observed to activate fibroblasts via TGF-β1 mRNA [13]. Furthermore, exosomes secreted by endothelial cells derived from human induced pluripotent stem cells have been shown to enhance myocardial infarction recovery in mice [14], indicating a significant role of exosomes in both local and remote cell-cell signaling. Exosomal proteins, miRNAs, DNAs, metabolites, and RNAs play pivotal roles in intercellular communication, influencing recipient cells through paracrine and autocrine pathways. miRNAs are recognized as significant mediators in these processes and represent promising candidates for disease therapy [15]. Cardiac miRNAs, such as miR-1, miR-328, miR-26, and miR-133a, are known to regulate ion channel genes that control cardiac rhythmicity, conduction, and automaticity [16–20]. miRNAs from either endothelial cells or cardiomyocytes may contribute to the regulation of endothelial or cardiac function.

We proposed that exosome shedding by endothelial cells exposed to catecholamine may be altered, leading to dysfunction in both endothelial cells and cardiomyocytes. This study aimed to verify our hypotheses with three specific objectives: (1) to determine whether exosomes from endothelial cells challenged with epinephrine (Epi) influence cardiomyocytes, (2) to assess Epi-effects on the expression levels of miRNAs in human coronary microvascular endothelial cells (HCMECs) and their exosomes, and (3) to decipher the mechanisms by which endothelial exosomes impact cardiomyocytes in the context of TTC. Our experimental findings reveal that exosomes secreted by endothelial cells challenged by high concentrations of Epi play a crucial role in modulating the electrophysiological properties of human-induced pluripotent stem cell-derived cardiomyocytes (hiPSC-CMs). Exosomal miR-126-3p secreted by endothelial cells significantly downregulates the expression of regulator of G protein signaling 3 (RGS3) as a molecular mechanism for Epi-induced abnormal electrophysiological properties and arrhythmia of cardiomyocytes.

## Materials and methods

### Serum from healthy donors and TTC patients

Written informed consent was obtained from all subjects or their families in accordance with the Declaration of Helsinki. Diagnosis was based on the final diagnosis at discharge, determined by the treating physician. Diagnosis of TTC was made based on previously described criteria [21]. Healthy control subjects were selected based on the absence of altered coronary arteries in coronary angiography diagnostics. Blood samples were collected from patients diagnosed with acute TTS during their hospitalization. For patients who had recovered from TTS, blood samples were obtained at a follow-up visit several months after the acute episode. At the follow-up, the patients were asymptomatic.

The blood was drawn from healthy donor subjects, TTC patients, and TTC-recovered patients, followed by incubation at room temperature for 60 min. Subsequently, the blood samples were centrifuged at 1500× g for 15 min to isolate the serum, which was then immediately frozen and stored at -80 °C until extraction. Serum samples were individually quantified and analyzed.

### Epinephrine level in serum

Epi concentration in serum was measured using a human EPI (Epinephrine/Adrenaline) ELISA Kit (AssayGenie, UNFI0039) following the manufacturer’s instructions. Briefly, 50 µL of Standard, Blank, or Sample, along with 50 µL of biotin-labeled antibody working solution were added to each well. The plate was then covered with the provided sealer and gently tapped to ensure thorough mixing. Subsequently, it was incubated for 45 min at 37 °C. Following a wash step, 100 µL of SABC working solution was added to each well and incubated for an additional 30 min at 37 °C. Afterward, 90 µL of TMB Substrate and 50 µL of Stop Solution were sequentially added to each well. The wells turned yellow immediately. Finally, the optical density (OD Value) of each well was determined simultaneously using a microplate reader set to 450 nm.

### Generation and culture of human-induced pluripotent stem cells-derived cardiomyocytes (hiPSC-CMs)

Human iPSCs were generated from healthy donor peripheral mononuclear blood cells. The hiPSC line UMGi130-A clone 5 (isWT11.5, here abbreviated as D5) was reprogrammed using integration-free Sendai virus with the reprogramming factors OCT4, SOX2, KLF4 and c-MYC, and was applied previously [22]. Human iPSCs were maintained in TeSR™-E8™ medium (05990, StemCell Technologies) supplemented with TeSR™-E8™ 25× supplement. When hiPSCs reached 70–80% confluence in a T-75 flask coated with Matrigel (Corning, 354230) at a 1:100 dilution, they were differentiated into cardiomyocytes with the cardiac differentiation medium including RPMI 1640 Medium with GlutaMAX™ Supplement (61870044, Thermo Fisher Scientific), 1% Pen/Strep (15140-122, Thermo Fisher Scientific), 1% Sodium Pyruvate (11360-039, Thermo Fisher Scientific), 2% B27 (17504001, Thermo Fisher Scientific), 0.2 mg/ml L-ascorbic acid 2-phosphate (A8960, Sigma-Aldrich) in a 24-well plate. On day 0, hiPSCs were treated with 6 µM CHIR99021 (130-103-926, StemMACS). On day 2, 5 µM IWP-2 (72122, StemCell Technologies) was used to treat hiPSCs. On day 4, the cardiac differentiation medium was used to feed the cells. From day 12 to day 14, the cells were fed with the cardio-selection medium containing RPMI 1640 (without glucose and glutamine) supplemented with 5 mM Sodium DL-Lactate Solution (BCBQ6934V, Sigma Aldrich), 50 mM 2-mercaptoethanol (31350010, Gibco). hiPSC-CMs are cultured for 50–60 days and used for biological studies or digested into single cells for patch clamp measurements.

### Culture of human cardiac microvascular endothelial cells (HCMECs)

HCMECs were purchased from PromoCell (Heidelberg, Germany) and were cultured according to the manufacturer’s instructions. The cells were grown in Endothelial Cell Growth Medium MV2 (EMV2) supplemented with SupplementMix (C-22022, PromoCell). HCMECs were cultured in matrigel coated flask. The medium was changed every other day until they reached 90% confluence before experiments.

After reaching approximately 80% confluence, HCMECs were transfected with miR-126-3p mimic (5 µl, 20 µM), miR-126-3p inhibitor (5 µl, 20 µM) and miR-126-3p-NC (negative control) using Lipofectamine 2000 reagent (11668030, Thermo Fisher Scientific) in strict accordance with the manufacturer’s instructions. After transfection for 48 h, HCMECs were used for the downstream research.

### Exosomes isolation

The exosome secreted by HCMECs was isolated by ultracentrifugation as previously described [23]. Briefly, supernatants from HCMECs and Epi-treated HCMECs incubated with exosome-free serum for 48 h were collected and subjected to gradient centrifugation at 200 g for 10 min, 2,000 g for 20 min, and 10,000 g for 30 min. The resultant supernatant was further centrifuged at 100,000 g for 70 min to precipitate the exosomes. Afterwards, the precipitate was washed with PBS and centrifuged at 100,000 g for 70 min to harvest the exosomes. Exosomes and Epi-treated exosomes (Epi-exo) were stored at -80 ºC for long-term preservation. Exosomes (20 µg) were directly applied to hiPSC-CMs and incubated for 48 h for subsequent experiments.

### Characterization of exosomes by Western blot and nanoparticle tracking analysis (NTA)

Exosomes were characterized utilizing specific CD9 antibody (PA5-11559, ThermoFisher scientific), CD81 antibody (MA5-13548, ThermoFisher scientific), GM130 antibody (sc-55591, Santa Cruz), CD63 antibody (Ts63) (10628D, ThermoFisher scientific), GRP94 antibody (ab13509, Abcam) through Western blot analysis. Subsequently, 1 µl of concentrated exosomes was diluted in 1 ml of sterile filtered PBS, and the size and concentration of exosomes were quantified in real-time using a ZetaView instrument (Particle Metrix, Meerbusch, Germany).

### Identification of exosomes by flow cytometry

Exosomes extracted from the HCMECs culture medium were stained for flow cytometry as per the guidelines provided by ExoStep Cell Culture (ExoS-25-C9, Immunostep). Initially, 10–15 µg of exosomes were introduced into a 12 × 75 mm cell counting tube and gently agitated using a pipette. Subsequently, they were left to incubate in darkness at room temperature overnight. Following this incubation, the exosomes bound to beads were subjected to a washing step employing 1 ml of a 1× assay buffer. The tube containing the mixture was then placed on a magnetic rack for 5 min to facilitate the collection of magnetic beads. The supernatant was aspirated from the tubes, and then 5 µl of the primary detection antibody was added to the tube containing the bead-bound exosomes. The mixture was then incubated in darkness for 60 min at 2–8ºC. Subsequently, the magnetic beads were collected, and the supernatant was discarded. The remaining sample was resuspended in 350 µl of 1× assay buffer and immediately analyzed using a flow cytometer. Exosomes were quantified using the BD FACS Canto™ II (Becton Dickinson, Heidelberg, Germany), and data analysis was performed using BD FACS Diva software (Version 8.0.1).

### Immunofluorescence staining

The growth medium was discarded from the hiPSC-CMs slides and subjected to three washes with PBS. Subsequently, the hiPSC-CMs on all slides were fixed with 4% paraformaldehyde (P087.4, Carl Roth) at room temperature for 10 min in the absence of light. After fixation, the cells were permeabilized with 0.1% triton X-100 (3051.3, Carl Roth) for 10 min, followed by three additional washes with PBS. The samples were blocked with 5% bovine serum albumin (BSA, Sigma-Aldrich) in PBS at room temperature for 1 h. The cells were then incubated overnight at 4 °C with recombinant Anti-Cardiac Troponin T antibody [EPR20266] (ab209813, Abcam). The following day, hiPSC-CMs were treated with Goat anti-Rabbit IgG (H + L) Cross-Adsorbed Secondary Antibody, Alexa Fluor 488 (A-11008, Thermo Fisher Scientific) for 1 h at room temperature in the dark. Afterward, DAPI (Biozol) was added for nuclear staining, and the cells were incubated for 10 min at room temperature in the dark.

Immunofluorescence staining of human iPSCs was measured using a pluripotent stem cell 4-marker immunocytochemistry kit (A24881, ThermoFisher Scientific) following the manufacturer’s instructions. In brief, cultured hiPSCs were fixed with 4% paraformaldehyde in 1×PBS and incubated overnight at 4 °C with antibodies against SSEA-4, OCT4, SOX2, or TRA-1-60. After a brief wash with 1×PBS, corresponding secondary antibodies were applied for 1 h at room temperature. Nuclei were stained with NucBlue™ Fixed Cell Stain (DAPI). The results were visualized using the TCS SP-8 upright confocal microscope (Leica, Germany) with a Plan-Apochromat 40×/0.6 objective. All other antibodies used for hiPSC characterization are listed in Table S1.

### Assessment of endothelial cell-derived exosome labeling

hiPSC-CMs in 24-well plates were digested into single cells as previously described [24]. Exosome uptake by cardiomyocytes was tracked using the PKH26 Red Fluorescent Cell Linker Kit for General Cell Membrane Labeling (PKH26GL-1KT, Sigma-Aldrich). Exosomes isolated from HCMECs supernatant and 4 µl of PKH26 or 4 µl of PBS were mixed up with 0.5 mL of Diluent C, followed by incubation for 5 min at room temperature in the dark. To halt over-staining, 2 ml of FBS was added. The labeled exosomes were then centrifuged at 110,000 × g for 1 h to eliminate excess dye, and the exosome pellet was resuspended in 200 µl of PBS. Subsequently, 20 µg of labeled exosomes, PBS, and unlabeled exosomes were added to hiPSC-CMs and incubated for 8 h at 37 °C in the dark. Exosome uptake by cardiomyocytes was then visualized using the TCS SP-8 upright confocal microscope (Leica, Germany) with a Plan-Apochromat 40×/0.6 objective.

### Single-cell contraction measurement

Single hiPSC-CM contraction was measured by a single-cell contraction measuring system (MyoCam-S IonOptix). The contraction of spontaneously beating hiPSC-CMs was monitored at 37 °C. Both rhythmic and arrhythmic events were evaluated under conditions with and without drug treatment. The data were analyzed using MyoCam-S IonOptix software.

### Patch clamp experiment

Action potentials (APs) and membrane currents were measured in hiPSC-CMs using the standard whole-cell patch-clamp technique in both current- and voltage-clamp modes. Patch electrodes were fabricated from thin-walled borosilicate glass capillaries (TW150F-4, World Precision Instruments) and pulled using the DMZUniversal Puller (Zeitz-InstrumenteVertriebs GmbH, Martinsried, Germany). The pipette resistance was between 2 and 4 MΩ. To induce action potentials, pulses of 800–1000 pA lasting approximately 5 ms were applied in the current-clamp mode. Experiments were conducted at room temperature (22–25 °C). APs were recorded at a frequency of 1 Hz in a bath solution composed of (mM): 5.9 KCl,130 NaCl, 1.2 MgCl_2_, 2.4 CaCl_2_, 10 HEPES, and 11 Glucose (pH 7.4, adjusted with NaOH). The pipette solution consisted of (mM): 1 MgCl_2_, 20 KCl, 2 ATP, 110 K-aspartate, 0.5 EGTA, 10 HEPES, and 0.5 GTP (pH 7.2, adjusted with KOH). Key parameters such as resting membrane potential (RP), maximal depolarization velocity (Vmax), action potential duration at 10%, 50%, and 90% repolarization (APD10, APD50, APD90), as well as ion channel currents, were analyzed using FitMaster software.

Ion channel currents in hiPSC-CMs were recorded in voltage-clamp mode using specific protocols and solutions tailored for different currents. Specifically, the sodium current (I_Na_) was measured using an external solution consisting of (mM): 1.8 CaCl_2_, 20 NaCl, 110 CsCl, 10 HEPES, 1 MgCl_2_, 0.001 nifedipine, and 10 Glucose (pH 7.4 adjusted with CsOH). The internal pipette solution was composed of (mM): 5 EGTA, 10 NaCl, 2 CaCl2, 135 CsCl, 3 MgATP, 10 HEPES, and 2 TEA-Cl (pH 7.2 adjusted with CsOH).

To record the L-type calcium channel current (I_Ca−L_), the external solution comprised the following (in mM): 5 CaCl_2_, 140 TEA-Cl, 10 HEPES, 1 MgCl_2_, 0.02 TTX, 3 4-AP, and 0.003 E-4031, with the pH adjusted to 7.4 using CsOH. The internal electrode solution consisted of (in mM): 10 HEPES, 10 NaCl, 2 CaCl_2_, 135 CsCl, 2 TEA-Cl, 3 MgATP, and 5 EGTA (pH 7.2 adjusted with CsOH).

To record the Na^+^/Ca^2+^ exchanger current (I_NCX_), the extracellular solution consisted of the following components (in mM): 10 HEPES, 135 NaCl, 1 MgCl_2_, 10 CsCl, 2 CaCl_2_, 10 glucose, 0.1 niflumic acid, 0.01 nifedipine, 0.05 lidocaine, 0.02 dihydroouabain and 0.05 lidocaine (pH 7.4 (CsOH)). The intracellular solution contained (in mM): 10 NaOH, 150 CsOH, 5 EGTA, 75 aspartic acid, 1 MgCl_2_, and 2 CaCl_2_ (pH 7.2 (CsOH)).

To measure the current of the rapidly activating delayed rectifier channel (I_Kr_), Cs^+^ is used as the charge carrier in place of K^+^ ions. The bath solution was utilized (in mM): 2 MgCl_2_, 140 CsCl, 10 Glucose, and 10 HEPES (pH = 7.4 adjusted with CsOH). The internal solution comprised (in mM): 2 MgCl_2_, 140 CsCl, 10 EGTA, and 10 HEPES (pH 7.2 adjusted with CsOH).

To record the transient outward potassium current (I_to_) and slow delayed rectifier potassium current (I_Ks_), the external bath solution used contained (in mM): 10 HEPES, 5.9 KCl, 130 NaCl, 1.2 MgCl_2_, 2.4 CaCl_2_ and 11 Glucose (pH 7.4 (NaOH)). The internal solution comprised (in mM): 10 HEPES, 20 KCl, 0.5 EGTA, 110 K-aspartate, 2 ATP, 1 MgCl_2_, and 0.5 GTP (pH 7.2 (KOH)). Notably, to prevent interference from I_Na_, I_Ca−L_, and I_Kr_ during Ito recording, 10 µM TTX, 10 µM nifedipine, and 3 µM E-4031 were added to the bath solution. For I_Ks_ recording, the external solution was supplemented with 10 µM TTX, 10 µM nifedipine, and 3 mM 4-AP.

### Real-time fluorescence quantitative polymerase chain reaction (qPCR)

For mRNAs, total RNA was extracted from hiPSC-CMs using the RNeasy Mini kit (74106, Qiagen), followed by cDNA synthesis with the high-capacity cDNA reverse transcription kit (4368814, Thermo Fisher Scientific) following manufacturers’ recommendations. qPCR was conducted using the StepOne Plus Real-Time PCR platform (Applied Biosystems). The reactions were prepared with HotRox Master Mix (119405, BIORON).

miRNAs were isolated from exosomes or serum using the exoRNeasy Midi Kit (77144, Qiagen). TaqMan™ MicroRNA Reverse Transcription Kit (4366596, Thermo Fisher Scientific) was employed to convert miRNA into cDNA. Real-Time PCR technique was performed on a StepOnePlus device (Applied Biosystems) following the manufacturer’s protocol using TaqMan Fast Advanced Master Mix (4444557) and TaqMan probes (20×) for specific miRNAs (Assay name: hsa-miR-126-3p, Assay ID:002228; hsa-miR-16, Assay ID: 000391; hsa-miR-26a, Assay ID: 000405; hsa-miR-133a, Assay ID: 002246; hsa-RNU6B, Assay ID:001093, Thermo Fisher Scientific). Relative mRNA and miRNA expression levels were calculated as the expression of the target gene relative to GAPDH in samples, which was analyzed using the 2^−Δ(ΔCT)^ method, where ΔCT = CT target gene - CT_GAPDH_, and Δ(ΔCT) = ΔCT treated – ΔCT control. The gene-specific primer sequences employed in this study are provided in Tables S2 and S3.

### Detection of intracellular calcium transients

To measure intracellular calcium transients, hiPSC-CMs were loaded with the fluorescent calcium indicator Fluo-3 AM (F1242, Thermo Fisher Scientific). Initially, a 50 µg aliquot of membrane-permeable Fluo-3 AM was dissolved in 44 µl of a 20% w/v Pluronic F-127 stock solution in DMSO, creating a 1 mM Fluo-3 AM stock. The hiPSC-CMs were washed twice with pre-incubated PSS (described below). Subsequently, Fluo-3 AM stock solution (10 µl) was added to 1 ml of PSS to yield a 10 µM working solution of Fluo-3, which was used to incubate the cells for 10 min at room temperature, protected from light. Following this, the PSS was carefully aspirated, and the cells were rinsed 4–5 times with fresh PSS. Finally, the cells were maintained in PSS at room temperature for approximately 30 min to allow for de-esterification. After de-esterification, the fluorescence of the cells was measured by using the Cairn Optoscan calcium imaging system (Cairn Research, UK), with excitation at 488 nm and emission at 520 nm.

The PSS contained (mM): 1.2 MgCl_2_, 130 NaCl, 2.4 CaCl_2_, 5.9 KCl, 10 HEPES, and 11 Glucose (pH 7.4 with NaOH).

### Dual luciferase reporter assay

HEK293T cells (CRL-3216, ATCC) were cultured in Dulbecco’s Modified Eagle Medium (DMEM) supplemented with 1% penicillin-streptomycin (15140122, Thermo Fisher Scientific), 10% fetal calf serum (FCS), and 2 mM Lglutamine (25030123, Thermo Fisher Scientific) to study the mRNA silencing function of miR-126-3p. The recombinant vector of psiCHECK2 WT-RGS3 or psiCHECK2 MT-RGS3 was co-transfected with either miR-126-3p-mimic or miR-126-3p-NC (negative control) into HEK293T cells by Lipofectamine 2000 Transfection Reagent (11668030, Thermo Fisher Scientific). Cell lysates were made 48 h after transfection. Renilla and Firefly luciferase activity was quantified using the Dual Luciferase kit (Promega, USA) following the manufacturer’s protocol. Luciferase activity was detected by the Spark^®^ multimode microplate reader.

### Western blot

Total proteins were extracted from HEK293T cells, HCMECs, or hiPSC-CMs using 1 × RIPA lysis buffer supplemented with a Halt™ Protease and Phosphatase Inhibitor Cocktail (78440, Thermo Fisher). Equal amounts of proteins were loaded into each lane and separated by either 10% or 15% SDS-PAGE gels, then transferred onto PVDF membranes (Millipore, USA) (Millipore, USA). The membranes were incubated in 5% non-fat milk in TBST for 1 h at room temperature and subsequently with the corresponding primary antibodies overnight at 4 °C. The primary antibodies used were as follows: RGS3 Polyclonal Antibody (66790-1-Ig, Proteintech), mouse-anti-GAPDH (5G4MAb6C5, HyTest), GNAS (10150-2-AP, proteintech). On the next day, the membranes were washed three times for 10 min each in TBST and then incubated with the respective secondary antibodies for 60 min. The secondary antibodies employed were anti-Mouse IgG (Fab specific)-Peroxidase antibody produced in goat (A3682, Sigma-Aldrich) and anti-Rabbit IgG (whole molecule)-Peroxidase antibody produced in goat (A0545, Sigma-Aldrich). After three additional washes of 10 min each in TBST, the results were detected using chemiluminescence. The band intensities were then quantified using Image J software for analysis.

### Statistical analysis

All data are presented as mean ± SD and were analyzed using SigmaPlot 14.0 (Systat GmbH, Germany) and GraphPad Prism 7 (GraphPad Software Inc., California, USA). Unpaired Student’s t-test analysis was employed to compare two independent groups with a normal distribution, while one-way ANOVA followed by the Holm-Sidak post-test was utilized for comparisons involving more than two groups. The Fisher’s exact test was employed for comparisons of categorical variables. A p-value less than 0.05 was considered statistically significant.

## Results

### Isolation and characterization of exosomes from HCMECs

**Fig. 1:**
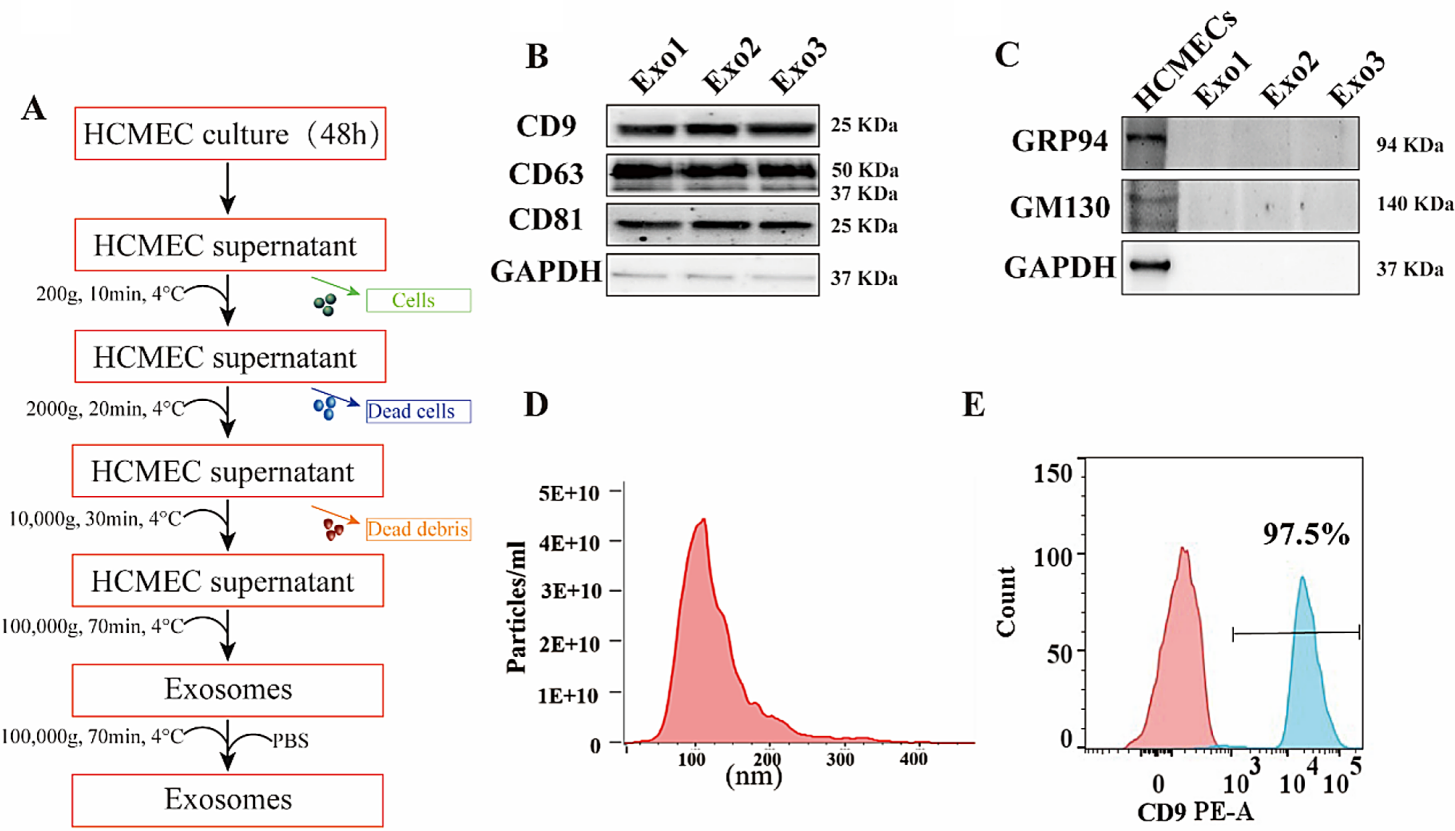
Isolation and characterization of exosomes from HCMEC culture supernatant. (**A**) Flow chart for obtaining exosomes from HCMECs supernatant by ultracentrifuge. (**B**) Western blot showing exosomal markers CD9 (~ 25 kDa), CD63 (between 37 and 50 kDa), and CD81 (~ 25 kDa) in exosomes (10 µg lysates) isolated from the supernatant of HCMECs of three samples (Exo 1, Exo 2, Exo 3). (**C**) GRP94 (~ 94 kDa) and GAPDH (~ 37 kDa) serve as a negative control and a loading control, respectively. (**D**) Size distribution of HCMECs-derived exosomes by ZetaView NTA analysis. (**E**) Percentage of CD9-positive exosomes displayed by flow cytometry

To investigate potential endothelial cell-derived factors implicated in catecholamine-induced cardiac dysfunction, exosomes were purified from the supernatant of cultured HCMECs through a series of differential centrifugation followed by ultracentrifugation (Fig. 1A). Western blot analysis confirmed the high expression of exosome markers CD9, CD63, and CD81 in the isolated exosomes (Fig. 1B). Notably, endoplasmic reticulum (ER) stress-related proteins such as glucose-regulated protein 94 (GRP94) and the Golgi marker GM130 were absent in exosomes but present in HCMECs, indicating the absence of cellular protein or intracellular debris contamination in exosomes (Fig. 1C). Nanoparticle Tracking Analysis (NTA) indicated that the average diameter of the HCMEC-derived exosomes was approximately 100 nm (Fig. 1D). Additionally, flow cytometry analysis revealed that 97.5% of vesicles were positive for CD9 (Fig. 1E). These findings collectively demonstrate the successful isolation and characterization of exosomes from HCMECs.

### Endothelial cell-derived exosomes can be taken up by hiPSC-CMs

**Fig. 2:**
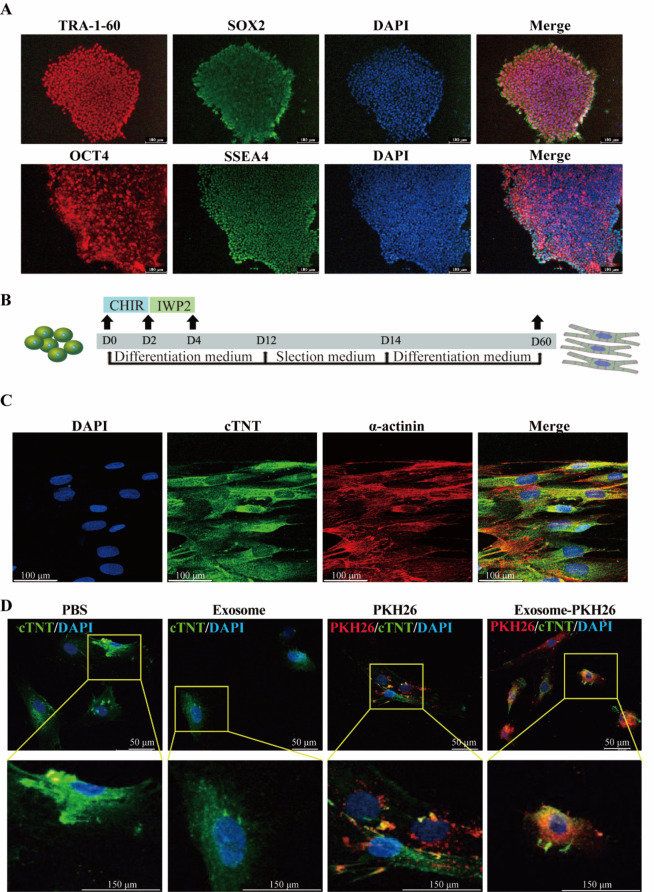
Exosomes secreted by endothelial cells can be taken up by hiPSC-CMs. (**A**) immunostaining of pluripotency markers of SSEA4 (green), OCT4 (red), SOX2 (green), and TRA-1-60 (red) with DAPI (blue) in hiPSCs. Scale bar: 100 μm for the left panels and 200 μm for the right panels. (**B**) Schematic representation of differentiating hiPSCs into cardiomyocytes. (**C**) Immunostaining of cardiac-specific marker alpha-actinin (red) and cTnT (green) with DAPI (blue) in hiPSC-CMs. Scale bar: 100 μm. (**D**) hiPSC-CMs treated with PBS, endothelial exosomes (Exo), PKH26 (red), and stained with cTnT (green). DAPI was used to stain cell nuclei (blue). Scale bars, 50 μm for the upper panels and 150 μm for the lower panels

hiPSCs, identified by the expression of pluripotency markers SSEA4, OCT4, SOX2, and TRA-1-60 (Fig. 2A), were differentiated into cardiomyocytes with a directed differentiation protocol (Fig. 2B). The successful differentiation of hiPSCs into cardiomyocytes was confirmed by the presence of cardiac troponin T (cTnT) and α-actinin (Fig. 2C). To investigate the potential impact of HCMEC-derived exosomes on hiPSC-CMs, the uptake of endothelial exosomes by hiPSC-CMs was evaluated. hiPSC-CMs were treated with either PBS, exosomes, or exosomes labeled with PKH26 for visualization. The results depicted in Fig. 2D demonstrate the uptake of HCMEC-derived exosomes by hiPSC-CMs, suggesting a potential role of exosomes in the functionality of hiPSC-CMs.

### Endothelial cell-derived exosomes modulated arrhythmic events and action potential changes triggered by epinephrine in hiPSC-CMs

**Fig. 3:**
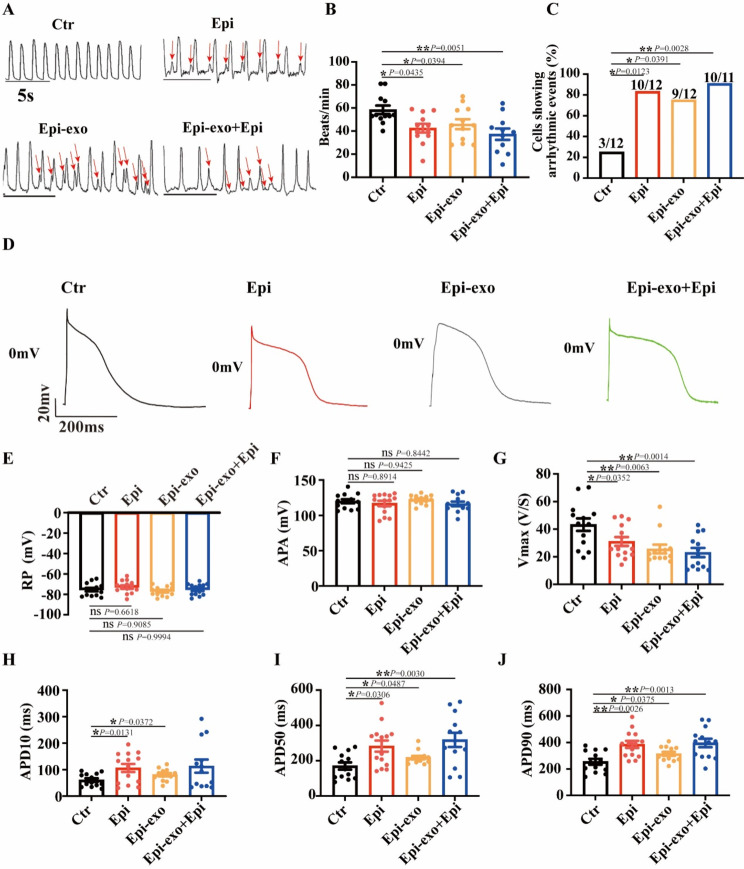
Endothelial cell-derived exosomes modulated arrhythmic events and action potential changes induced by epinephrine in hiPSC-CMs. hiPSC-CMs were treated with either vehicle (Ctr) or 500 µM epinephrine (Epi), or exosomes secreted by HCMECs treated with Epi (Epi-exo), or Epi-exo plus Epi (Epi-exo + epi), respectively. Action potential (AP) was measured by patch clamp whole cell recording with pulses of 1 nA for 5 ms at 1 Hz. (**A**) Representative traces of single-cell contractions in indicated groups (*n* = 11–12 cells). (**B**) Average values of beating rates of hiPSC-CMs in indicated groups (*n* = 11–12 cells). (**C**) Percentage of hiPSC-CMs with arrhythmia events in indicated groups. (**D**) Representative traces of APs in hiPSC-CMs of indicated groups. (**E**) Mean values of cell resting potential (RP) in indicated groups (*n* = 12–14 cells). (**F**) Mean values of action potential amplitude (APA) in indicated groups (*n* = 12–14 cells). (**G**) Mean values of maximal upstroke velocity of action potential (Vmax) in indicated groups (*n* = 12–14 cells). (**H**) Mean values of APD at 10% repolarization (APD10) in indicated groups (*n* = 12–14 cells). (**I**) Mean values of APD at 50% repolarization (APD50) in indicated groups (*n* = 12–14 cells). (**J**) Mean values of APD at 90% repolarization (APD90) in indicated groups (*n* = 12–14 cells). Data are shown as means ± SD. Scatter plots show the value of every measured cell. Numbers given in C represent the number of cells showing arrhythmic event/total number of measured cells. *P < 0.05, **P < 0.01, ***P < 0.001 determined by one-way ANOVA with Holm-Sidak post-hoc test or Fisher test

Elevated levels of epinephrine can induce endothelial dysfunction, characterized by impaired endothelial cell function and altered signaling pathways [25]. To assess the role of exosomes derived from dysfunctional endothelial cells in arrhythmia events, the occurrence of irregular or triggered beats, including delayed afterdepolarization (DAD)- or early afterdepolarization (EAD)-like events, was evaluated in spontaneously beating hiPSC-CMs. Notably, treatment with exosomes isolated from Epi-treated HCMECs (Epi-exo) significantly reduced the beating rate of hiPSC-CMs and induced episodes of arrhythmic events, including extra and irregular contractions, which is similar to the phenotype of TTC showing in Epi treated hiPSC-CMs (Fig. 3A-C). Importantly, the combination of Epi-exo with Epi treatment further reduced the beating frequency and exacerbated the irregular contractions in hiPSC-CMs (Fig. 3A-C). These findings suggest that endothelial exosomes derived from dysfunctional endothelial cells may contribute to stress-induced arrhythmias.

To investigate the mechanisms underlying arrhythmias triggered by high concentrations of Epi and the role of endothelial exosomes, we evaluated their effects on action potentials in hiPSC-CMs exposed to Epi. Our findings demonstrated that resting potential (RP) and action potential amplitude (APA) were not significantly affected by Epi, Epi-exo, or the combination of Epi and Epi-exo (Fig. 3D-F). However, Epi significantly decreased the maximum depolarization velocity (Vmax) and extended action potential duration at 10%, 50%, and 90% repolarization (APD10, APD50, APD90), respectively. These effects were mimicked by Epi-exo (Fig. 3D and G-J). Notably, Epi-exo alone replicated the effects of Epi. Furthermore, when Epi and Epi-exo were combined, there was a further decrease in Vmax and an additional prolongation of APD10, APD50, and APD90 (Fig. 3D and G-J). These findings suggest that exosomes from HCMECs may contribute to Epi-induced arrhythmic events by altering action potentials.

### Ionic mechanisms underlying action potential changes induced by Epi and exosomes

**Fig. 4:**
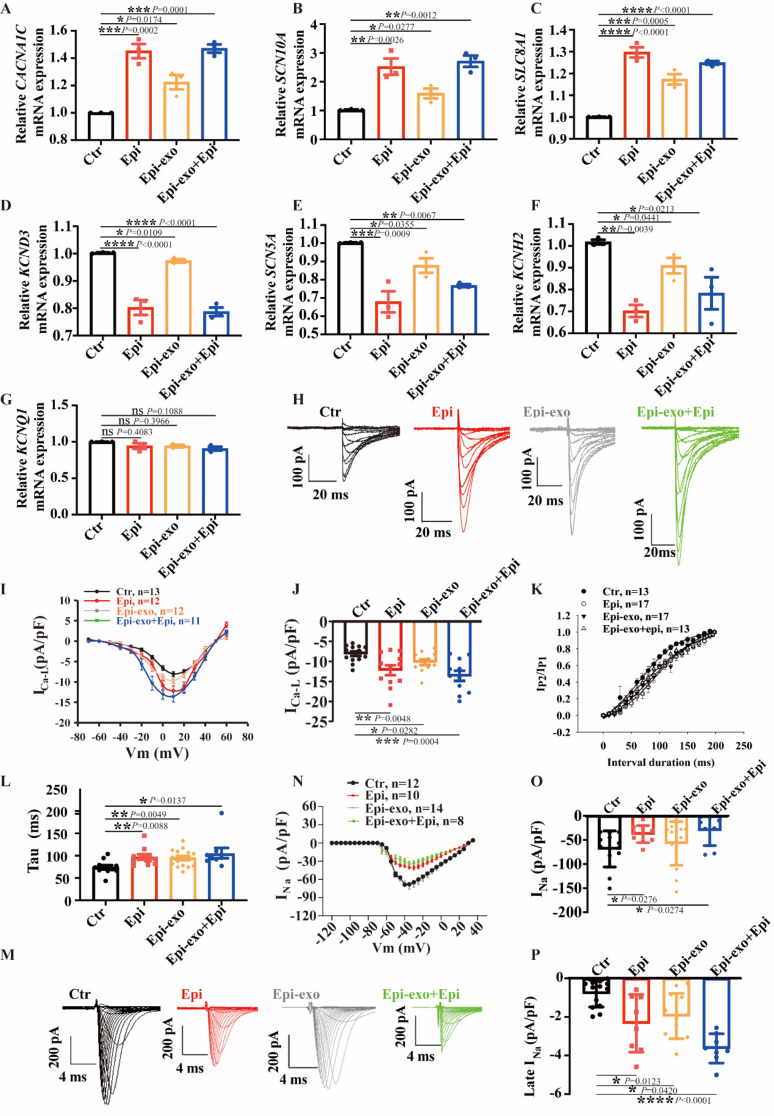
Changes of ion channel expression and currents induced by Epi and exosomes. hiPSC-CMs were treated with either vehicle (Ctr) or 500 µM epinephrine (Epi), or exosomes secreted by HCMECs treated with Epi (Epi-exo), or Epi-exo plus Epi (Epi-exo + epi), respectively. (**A**) Gene expression of *CACNA1C* in indicated groups (*n* = 3). (**B**) Gene expression of *SCN10A* in indicated groups (*n* = 3). (**C**) Gene expression of *SLC8A1* in indicated groups (*n* = 3). (**D**) Gene expression of *KCND3* in indicated groups (*n* = 3). (**E**) Gene expression of *SCN5A* in indicated groups (*n* = 3). (**F**) Gene expression of *KCNH2* in indicated groups (*n* = 3). (**G**) Gene expression of *KCNQ1* in indicated groups (*n* = 3). (**H**) Representative traces of I_Ca−L_ in indicated groups. (**I**) Current-voltage (I-V) relation curves of I_Ca−L_ in indicated groups. (**J**) Current density of I_Ca−L_ at 10mV in indicated groups (*n* = 11–13 cells). (**K**) Time course curves of recovery from inactivation in indicated groups. (**L**) Time constants (tau) of recovery from inactivation (*n* = 13–17 cells). (**M**) Representative traces of I_Na_ in indicated groups. (**N**) I-V curves of peak I_Na_ in indicated groups. (**O**) Current density of peak I_Na_ at -40 mV in indicated groups (*n* = 8–14 cells). (**P**) Current density of late I_Na_ at -40 mV in indicated groups (*n* = 8–14 cells). Data are presented as means ± SD. Scatter plots show the value of every experiment. *P < 0.05, **P < 0.01, ***P < 0.001 determined by one-way ANOVA with Holm-Sidak post-hoc test

To delve into the ionic mechanism underlying changes in action potentials induced by exosomes in the context of TTC, we examined the expression of key ion channels associated with action potential alterations using qPCR experiments. The results unveiled a significant increase in the expression of *CACNA1C* (encoding L-type calcium channel), *SCN10A* (encoding Nav1.8 sodium channel) and *SLC8A1* (encoding Na^+^/Ca^2+^ exchanger, NCX), but a decrease in *SCN5A* (encoding sodium channel, Nav1.5), *KCNH2* (encoding I_Kr_, also known as HERG channel, Kv11.1), and *KCND3* (encoding Ito channel, Kv4.3) genes upon Epi exposure (Fig. 4A-F). Notably, Epi effects were replicated by Epi-exo (Fig. 4A-G). However, neither Epi nor Epi-exo influenced the gene expression of *KCNQ1* (encoding I_Ks_ channels, Kv7.1) in hiPSC-CMs (Fig. 4G).

Subsequently, we used the corresponding protocol (Figure S1) to conduct voltage clamp experiments to measure various ion channel currents in hiPSC-CMs, including I_Ca−L_, I_Na_, late sodium current (I_Na−L_), Na^+^/Ca^2+^ exchanger current (I_NCX_), and potassium channel currents such as I_Kr_, I_to_, I_K1_and I_Ks_. Epi-Exo replicated the effect of Epi on I_Ca−L_ (Fig. 4H-J). Additionally, Epi-Exo combined with Epi shifted the steady-state activation curves towards more positive potentials (Figure S2A-B). Epi-exo shifted the steady-state inactivation curves towards more positive potentials (Figure S2C-D). Epi or Epi-exo changed the time constant of recovery from inactivation (Fig. 4K-L).

Epi reduced the peak sodium current (I_Na_), as depicted in Fig. 4M-O. Additionally, the combination of Epi-exo and Epi (Epi-exo + epi) notably shifted the steady-state activation curves towards more positive potentials, as demonstrated in Figures S3A and B. However, no significant alterations were observed in other biophysical properties of the peak sodium channel, such as voltage-dependent inactivation and time-dependent recovery from inactivation, in hiPSC-CMs across all experimental groups (Figure S3C-F). In contrast to the peak I_Na_, the late sodium current (I_Na−L_) was augmented by Epi, nevertheless, the epi-effect was also mimicked by Epi-Exo (Fig. 4P).

**Fig. 5:**
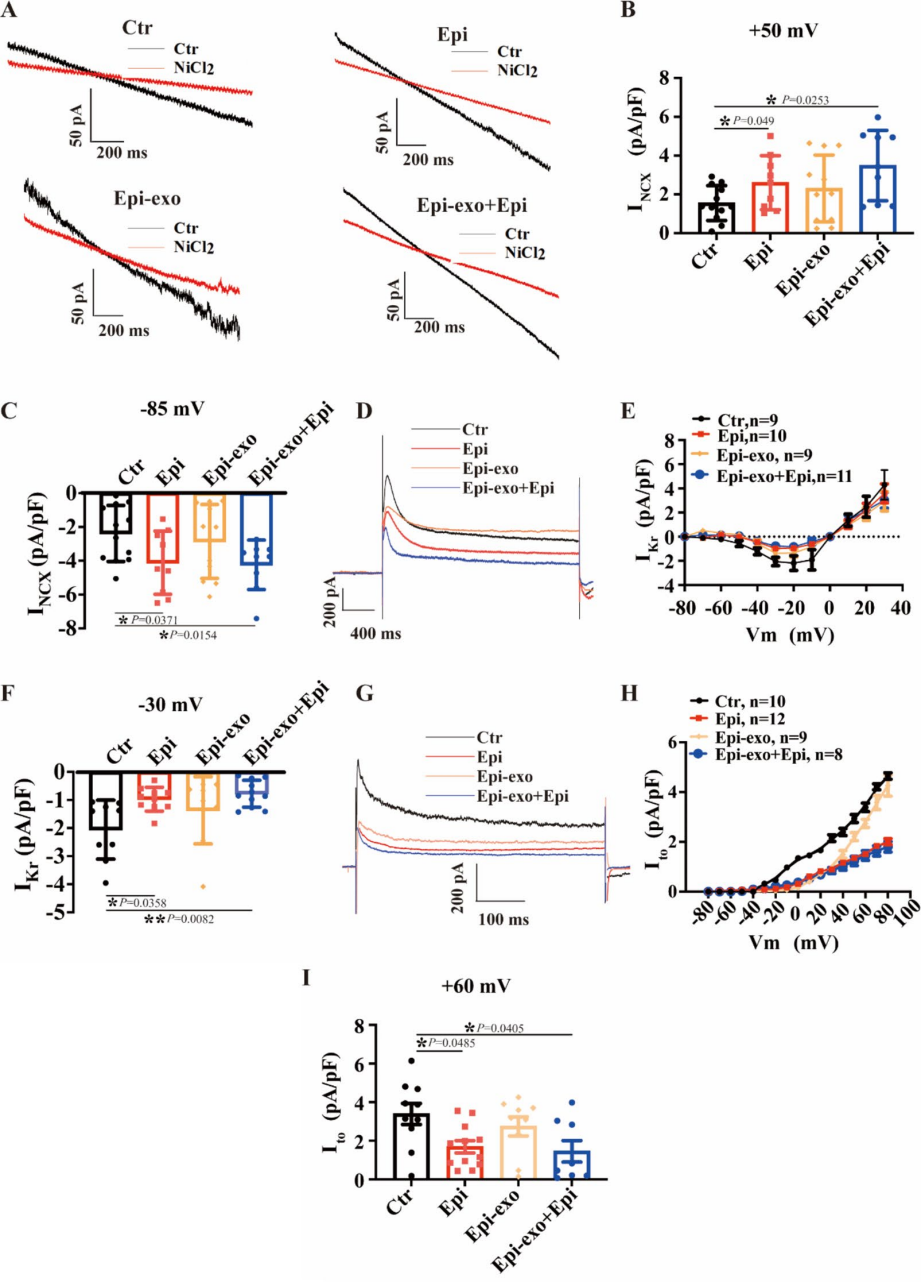
Effects of epinephrine and exosomes on INCX, IKr, Ito of hiPSC-CMs. hiPSC-CMs were treated with either vehicle (Ctr) or 500 µM epinephrine (Epi), or exosomes secreted by HCMECs treated with Epi (Epi-exo), or Epi-exo plus Epi (Epi-exo + epi), respectively. (**A**) Representative traces of INCX before and after application of 10 mM NiCl2, an INCX blocker, in hiPSC-CMs of indicated groups. (**B**) Current density of INCX at + 50 mV in indicated groups (*n* = 9–12 cells). (**C**) Current density of INCX at -85 mV in indicated groups (*n* = 9–12 cells). (**D**) Representative current traces of IKr at + 30 mV in hiPSC-CMs of indicated groups. (**E**) I-V relationship curves of IKr from − 80 mV to + 40 mV in indicated groups. (**F**) Current density of IKr at -30 mV in indicated groups (*n* = 9–11 cells). (**G**) The raw traces of Ito at + 60 mV in hiPSC-CMs in indicated groups. (**H**) I-V curves of 4-AP (Ito blocker)-sensitive currents in hiPSC-CMs of indicated groups. (**I**) Current density of Ito at + 60 mV in indicated groups (*n* = 8–12 cells). Data are shown as means ± SD. *P < 0.05, **P < 0.01, determined by one-way ANOVA with Holm-Sidak post-hoc tests

We also examined the involvement of I_NCX_ in modulating changes in action potentials induced by Epi or Epi-exo. We observed an increase in I_NCX_ in hiPSC-CMs treated with Epi compared to the control group at both + 50 mV and − 85 mV (Fig. 5A-C). Treatment with Epi-exo mimicked the effect of Epi on I_NCX_ at both + 50 mV and − 85 mV (Fig. 5A-C). Subsequently, we assessed the impact of Epi and Epi-exo on K^+^ channels. Epi notably diminished I_Kr_ and I_to_, replicated by Epi-exo (Fig. 5D-I). I_Ks_ remained unaffected by Epi or Epi-exo (Figure S4A-C).

### Mir-126-3p was involved in the effects of endothelial exosomes

**Fig. 6:**
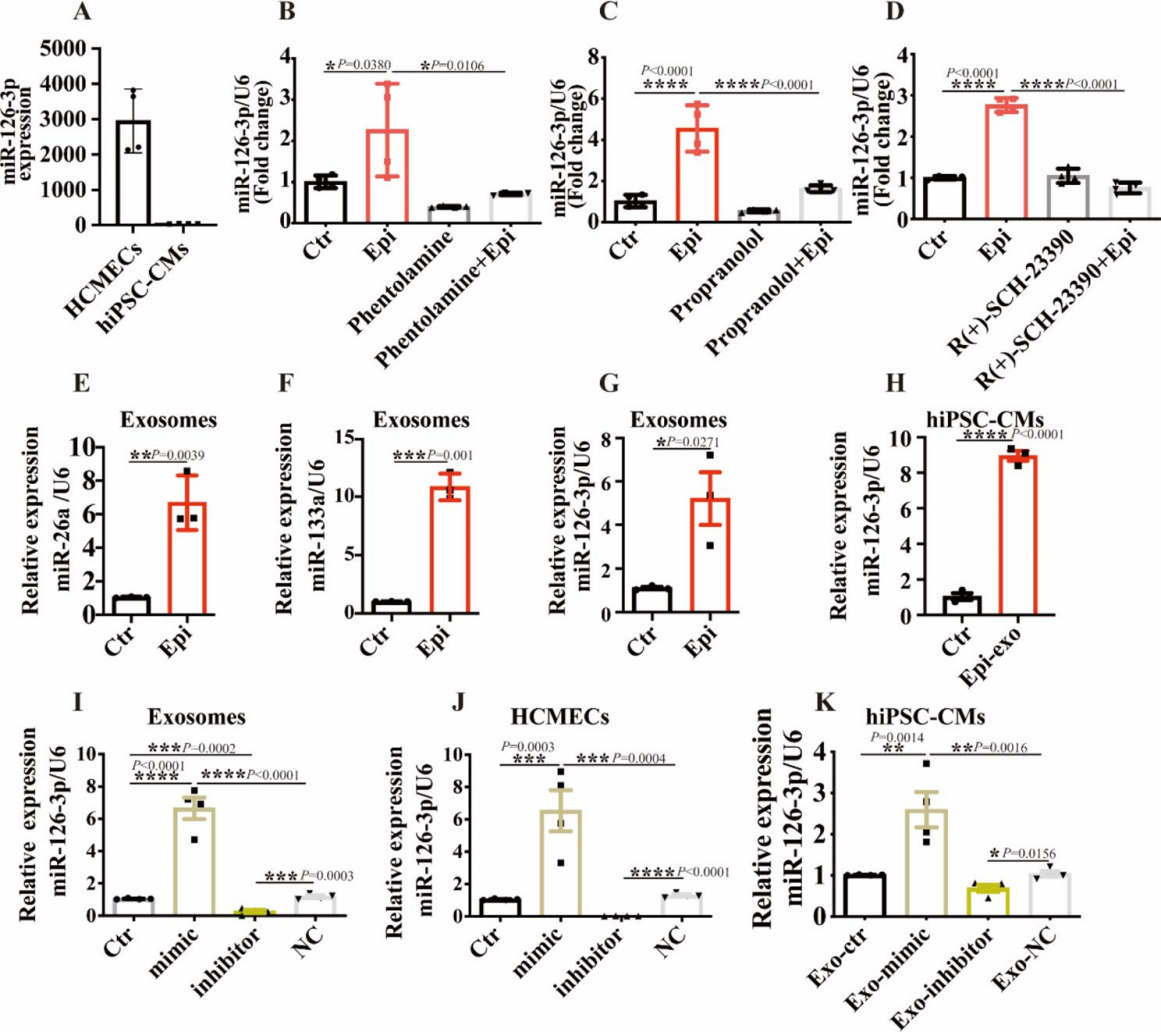
miR-126-3p was involved in the effects of endothelial exosomes. HCMECs were treated with vehicle (Ctr) or 500 µM epinephrine (Epi) and different miRNAs were measured in exosomes. Then, HCMECs were transfected with vehicle (Ctr) or miR-126-3p mimic (mimic) or miR-126-3p inhibitor (inhibitor) or miR-126-3p negative control (NC), respectively. Exosomes (Exo) were isolated from HCMECs of each group (Exo-ctr, Exo-mimic, Exo-inhibitor and Exo-NC) and applied to hiPSC-CMs. (**A**) miR-126-3p had a higher expression in HCMECs than hiPSC-CMs (*n* = 4). (**B**) α-adrenoceptor antagonist phentolamine inhibited the elevation of miR-126-3p levels induced by Epi in HCMECs (*n* = 4). (**C**) The effects of β-adrenoceptor antagonist propranolol on miR-126-3p levels in HCMECs (*n* = 4). (**D**) The effects of dopamine-adrenoceptor antagonist R(+)-SCH-23,390 on miR-126-3p levels in HCMECs (*n* = 4). (**E**) Epi increased the expression of miR-26a in Exo from HCMECs (*n* = 3). (**F**) Epi increased the expression of miR-133a in Exo from HCMECs (*n* = 3). (**G**) Epi increased the expression of miR-126-3p in Exo from HCMECs (*n* = 3). (**H**) The expression of miR-126-3p in hiPSC-CMs treated with Exo derived from HCMECs pretreated by Epi (Epi-exo) (*n* = 3). (**I**) The expression of miR-126-3p in Exo derived from HCMECs transfected with miR-126-3p mimic, miR-126-3p inhibitor, miR-126-3p NC (*n* = 4). (**J**) The expression of miR-126-3p in HCMECs transfected with miR-126-3p mimic, miR-126-3p inhibitor or miR-126-3p NC (*n* = 4). (**K**) The expression of miR-126-3p in hiPSC-CMs treated with Exo-ctr, Exo-mimic, Exo-inhibitor, Exo-NC (*n* = 4). Data are presented as means ± SD. Scatter plots show the value of every experiment. *P < 0.05, **P < 0.01, ***P < 0.001, ****P < 0.0001 determined by unpaired t-test or one-way ANOVA with Holm-Sidak post-hoc test

Given our findings indicating that exosomes derived from HCMECs exposed to Epi could replicate Epi-effects on ion channel function and arrhythmias in cardiomyocytes, we hypothesized that the Epi exposure altered the composition of endothelial cell-derived exosomes. Consequently, we conducted a detailed investigation to determine which component of endothelial cell-secreted exosomes modulated cardiomyocyte function. Prior research has linked miR-16, miR-26a, and miR-133a to TTC [26, 27]. Moreover, miR-126-3p, among the most crucial endothelial cell-restricted miRNAs, has been demonstrated to regulate vascular integrity and developmental angiogenesis [28, 29]. Hence, we assessed the expression of miR-26a, miR-133a, and miR-126-3p in exosomes derived from HCMECs treated with either vehicle or Epi. Our results showed miR-126-3p had a high expression in HCMECs, not hiPSC-CMs (Fig. 6A). Importantly, we observed: (1) Epi increased miR-126-3p expression in HCMECs, and this effect could be mitigated by α, β and dopamine receptor blockers (Fig. 6B-D); (2) the expression of miR-126-3p in Epi-exo was also increased (Fig. 6G); (3) application of Epi-exo to hiPSC-CMs led to miR-126-3p expression in hiPSC-CMs (Fig. 6H). In addition, we observed an upregulation of miR-26a and miR-133a in Epi-exo (see Fig. 6E-F). Given the limited research on miR-126-3p in TTC, we prioritized its investigation in our subsequent studies.

To investigate the role of miR-126-3p carried by exosomes in hiPSC-CMs, we manipulated the levels of miR-126-3p in exosomes using both mimic and inhibitor techniques. Specifically, miR-126-3p-mimic, miR-126-3p-inhibitor, or miR-126-3p-negative control (NC) were transfected into HCMECs to assess the expression of miR-126-3p in both cells and exosomes. Notably, miR-126-3p-mimic significantly augmented miR-126-3p expression in exosomes derived from HCMECs (Fig. 6I) as well as within the HCMECs (Fig. 6J) compared to the control (vehicle) group. Conversely, miR-126-3p-inhibitor markedly reduced miR-126-3p levels in both exosomes (Fig. 6I) and HCMECs (Fig. 6J) compared to the control group. No significant difference was observed in miR-126-3p expression between the miR-126-3p-NC group and the control group in either exosomes or HCMECs (Fig. 6I-J). Subsequent treatment of hiPSC-CMs with exosomes derived from HCMECs transfected with miR-126-3p mimic (Exo-mimic), miR-126-3p inhibitor (Exo-inhibitor), or miR-126-3p negative control (Exo-NC) revealed that Exo-mimic increased miR-126-3p expression in hiPSC-CMs, whereas Exo-inhibitor decreased miR-126-3p expression in hiPSC-CMs (Fig. 6K). These findings suggest that the Epi-induced elevation of miR-126-3p may contribute to the observed effects of endothelial exosomes on ion channels in hiPSC-CMs.

**Fig. 7:**
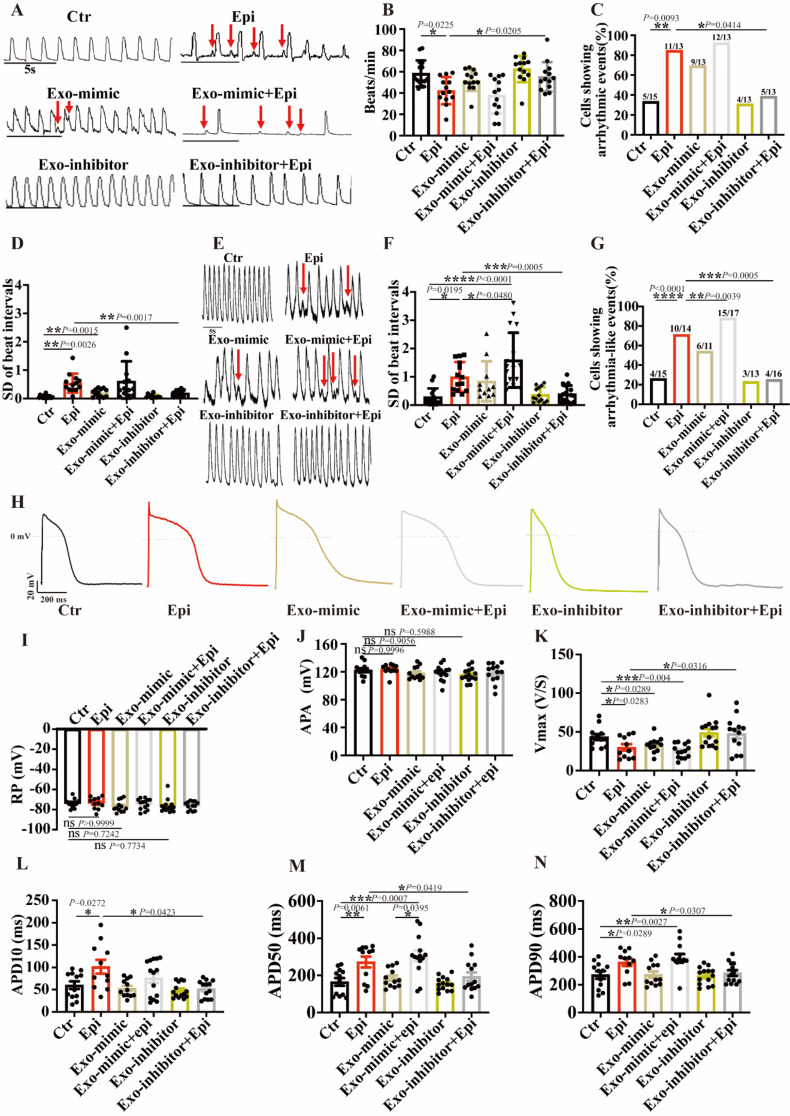
miR-126-3p could contribute to arrhythmic phenotype of hiPSC-CMs. hiPSC-CMs were treated with vehicle (Ctr) or 500 µM epinephrine (Epi) or exosomes from HCMECs transfected by miR-126-3p mimic (Exo-mimic), or Exo-miR-126-3p mimic plus Epi (Exo-mimic + epi), or exosomes from HCMECs transfected by miR-126-3p inhibitor (Exoinhibitor), or Exo-miR-126-3p inhibitor plus Epi (Exo-inhibitor + epi), respectively. (**A**) Contraction traces of single hiPSC-CMs in indicated groups. Arrows indicate EAD- or DAD-like events. (**B**) The effects of Exo derived from HCMECs transfected with miR-126-3p mimic or inhibitor on beat frequency (*n* = 13–15 cells). (**C**) The percentage of hiPSC-CMs showing arrhythmia events (*n* = 13–15 cells). (**D**) SD of beat intervals in indicated groups (*n* = 13–15 cells). (**E**) Calcium transient traces obtained in spontaneously beating hiPS-CMs of indicated groups. Arrows indicate EAD- or DAD-like events. (**F**) Standard deviation (SD) of beat intervals in indicated groups (*n* = 11–17 cells). (**G**) The percentage of hiPSC-CMs showing arrhythmia events in indicated groups (*n* = 11–17 cells). (**H**) Representative traces of AP recorded in hiPSC-CMs in indicated groups. (**I**) Mean values of resting potential (RP) in indicated groups. (**J**) Mean values of amplitude of action potential (APA) in indicated groups (*n* = 11–14 cells). (**K**) Mean values of maximal depolarization velocity (Vmax) in indicated groups (*n* = 11–14 cells). (**L**) Mean values of action potential duration at 10% repolarization (APD10) in indicated groups (*n* = 11–14 cells). (**M**) Mean values of action potential duration at 50% repolarization (APD50) in indicated groups (*n* = 11–14 cells). (**N**) Mean values of action potential duration at 90% repolarization (APD90) in indicated groups (*n* = 11–14 cells). Data are presented as means ± SD. Scatter plots show the value of every measured cell. *P < 0.05, **P < 0.01, ***P < 0.001, ****P < 0.0001 determined by one-way ANOVA with Holm-Sidak post-hoc test or Fisher-test

We then assessed the arrhythmogenic potential of hiPSC-CMs treated with Epi, either with or without Exo-mimic or Exo-inhibitor. Regarding the incidence of arrhythmia in spontaneously contracting hiPSC-CMs, Exo-mimic exerted effects similar to Epi (Fig. 7A-D and E-G). Conversely, Exo-inhibitor notably ameliorated Epi effects on parameters of hiPSC-CM contraction and calcium transients (Fig. 7A-G). These findings align with the above-described effects of Epi-exo in single-cell contraction assessments, supporting the conclusion that endothelial cell-released exosomes can modulate cardiomyocyte rhythm through miR-126-3p.

Thereafter, we conducted current clamp experiments to record the AP in hiPSC-CMs treated with Exo-mimic or Exo-inhibitor, both in the presence and absence of Epi. The data showed that neither Epi nor Exo-mimic had a significant impact on RP and APA (Fig. 7H-J). However, intriguingly, Exo-inhibitor blocked the effects of Epi on Vmax, APD50, and APD90 although Exo-mimic only replicates the Epi effect on Vmax (Fig. 7H, K and L-N). These findings suggest that miR-126-3p may play a role in mediating the effects of Epi on the electrophysiological properties of hiPSC-CMs. Therefore, further studies were carried out to explore the influence of miR-126-3p on ion channel expression in hiPSC-CMs. Exo-mimic significantly upregulated the mRNA levels of *CACNA1C*, *SCN10A*, and *SLC8A1* compared to exosomes derived from non-transfected HCMECs (Exo-ctr) or those transfected with Exo-NC. Conversely, Exo-mimic downregulated the mRNA expression of *SCN5A*, *KCND3*, and *KCNH2* (Figure S5). These data are principally consistent with results in those channel current measurements. Exo-inhibitor exerted effects opposite to Exo-mimic (Figure S5), further confirming the roles of miR-126-3p in the ion channel regulation.

### RGS3 is targeted by mir-126-3p in hiPSC-CMs

**Fig. 8:**
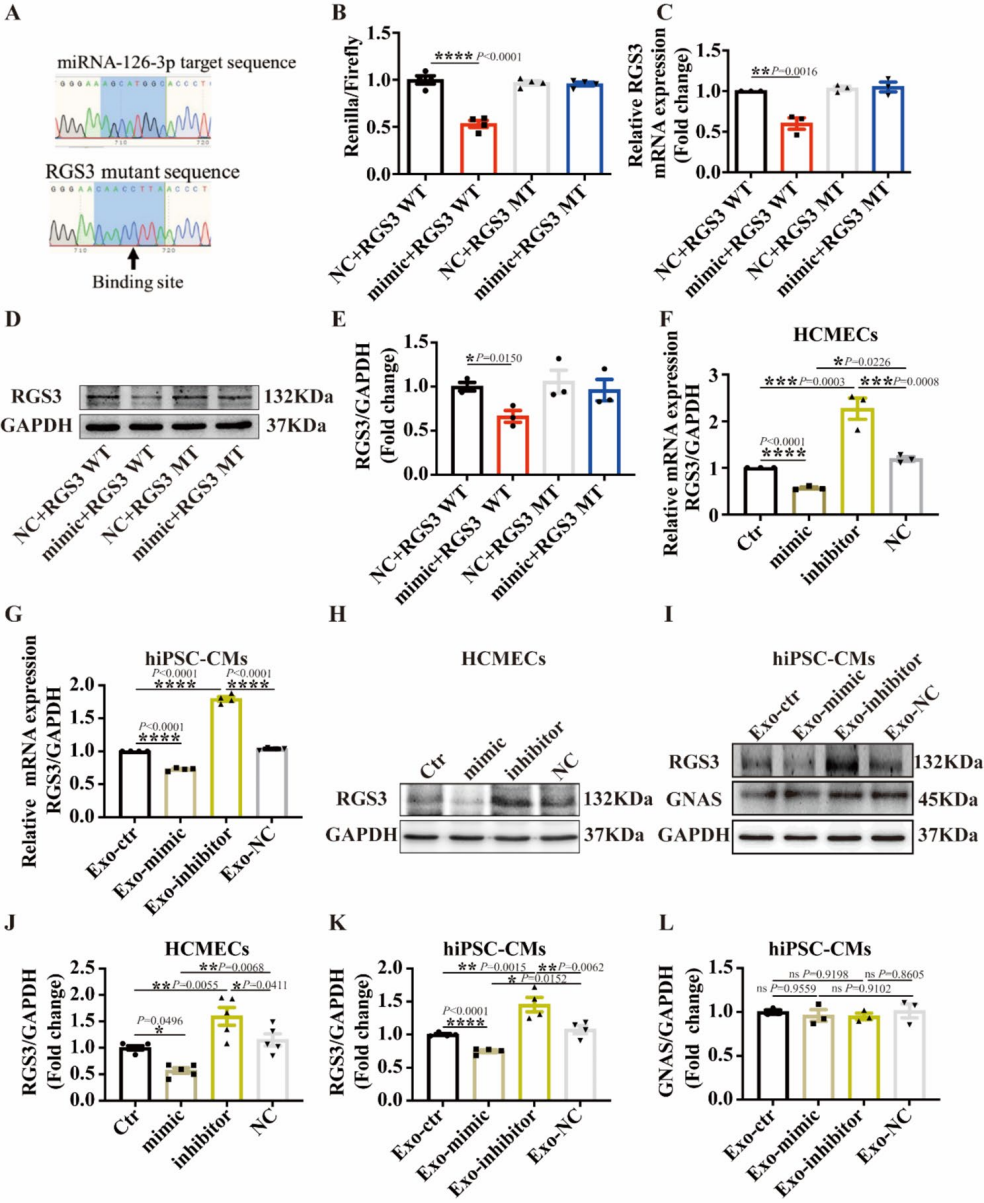
RGS3 is targeted by miR-126-3p in hiPSC-CMs. (**A**) Predicted miR-126-3p target sequence in RGS3-3’ UTRs by Target Scan and miRDB (https://www.targetscan.org/vert_80/ and https://mirdb.org/). Predicted target sequences of RGS3-3’UTRs were mutated for examining the change of binding to miR-126-3p. (**B**) Luciferase reporter assay of HEK293T cells transfected with RGS3-3’ UTR-WT (RGS3 WT) or RGS3-3’ UTR mutant (RGS3 MT) together with miR-126-3p NC or miR-126-3p mimic (*n* = 4). (**C**) The real time qPCR analysis of HEK293T cells transfected with RGS3 WT or RGS3 MT together with miR-126-3p NC or miR-126-3p mimic (*n* = 3). (**D**-**E**) Representative (**D**) and mean values (**E**) of western blot analyses of RGS3 protein level in HEK293T cells transfected with RGS3WT or RGS3 MT together with miR-126-3p NC or miR-126-3p mimic (*n* = 3). (**F**) The relative mRNA expression of RGS3 detected by qPCR in HCMECs transfected with vehicle or miR-126-3p mimic or miR-126-3p inhibitor or miR-126-3p NC (*n* = 3). (**G**) qPCR analysis of RGS3 mRNA level in hiPSC-CMs treated with Exo-ctr, Exo-mimic or Exo-inhibitor or Exo-NC (*n* = 4). (**H** and **J**) Representative bands and mean values of western blot analyzed RGS3 protein levels in HCMECs treated with miR-126-3p mimic or miR-126-3p inhibitor or miR-126-3p NC (*n* = 5). (**I** and **K**) Representative bands and mean values of RGS3 protein expression in hiPSC-CMs treated with Exo-ctr, Exo-mimic or Exo-inhibitor or Exo-NC measured by Western blot (*n* = 4). (**L**) Gs (GNAS) protein expression in hiPSC-CMs treated with Exo-ctr, Exo-mimic or Exo-inhibitor or Exo-NC measured by Western blot (*n* = 3). Results are presented as means ± SD. Scatter plots show the value of every experiment. *P < 0.05, **P < 0.01, ***P < 0.001, ****P < 0.0001 determined by one-way ANOVA with Holm-Sidak post-hoc test

To elucidate the potential mechanisms underlying the effects of miR-126-3p, we sought to identify its putative targets. The computational analyses utilizing TargetScan and miRDB resources (https://www.targetscan.org/vert_80/ and https://mirdb.org/) identified RGS3 as a promising target for miR-126-3p (Fig. 8A). Notably, miR-126-3p exhibited a specific binding site within the 3’-UTR of RGS3. To validate the regulatory interaction between miR-126-3p and RGS3, we conducted a dual luciferase reporter gene assay in HEK293T cells. Overexpression of miR-126-3p led to a suppression of luciferase activity associated with the 3’-UTR of RGS3-wild-type (WT) (Fig. 8A and B). Conversely, no significant alteration in luciferase activity was observed upon transfection with 3’-UTR RGS3 mutant (RGS3 MT) (Fig. 8A and B), suggesting specific targeting of RGS3 by miR-126-3p. Subsequent Western blot and qPCR analyses corroborated these findings, demonstrating that the miR-126-3p mimic attenuated both gene expression and protein levels of RGS3 in the RGS3 WT group (Fig. 8C-E).

To further examine the regulatory role of miR-126-3p on RGS3, we conducted both qPCR and Western blot analyses to assess its impact on the mRNA and protein expression of RGS3 in HCMECs and hiPSC-CMs. The expression of the RGS3 gene decreased in HCMECs treated with the miR-126-3p mimic and hiPSC-CMs treated with Exo-mimic, while its mRNA levels were upregulated in both types of cells treated with the miR-126-3p inhibitor (Fig. 8F-G). At the protein level, similar effects were detected (Fig. 8H-I). These findings collectively confirm RGS3 as a target of miR-126-3p. Of note, neither Exo-mimic nor Exo-inhibitor affected Gs (GNAS) protein expression (Fig. 8J).

### The mir-126-3p level in the serum of TTC patients is elevated

**Fig. 9:**
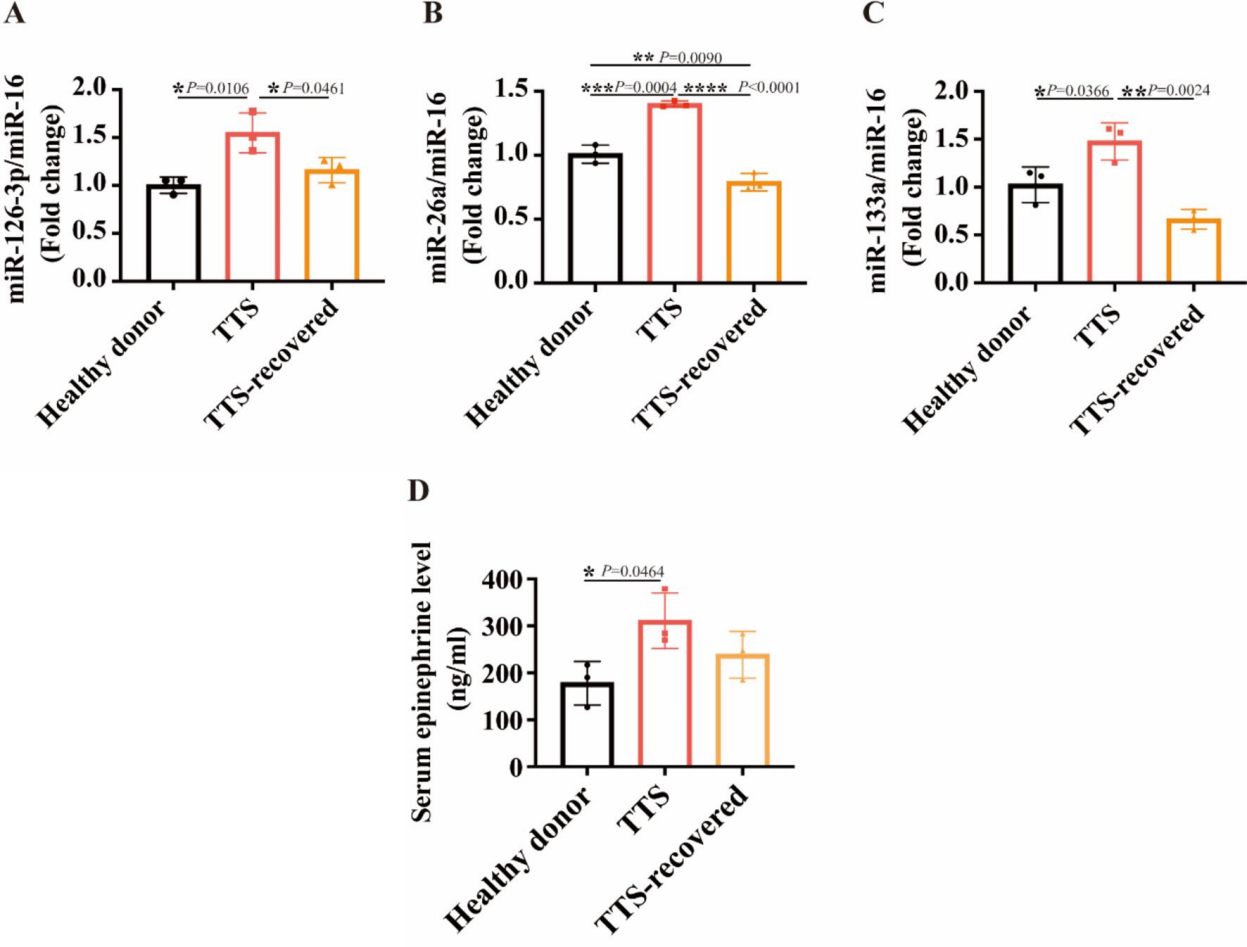
The miR-126-3p and epinephrine level in the serum of TTC patients is elevated. (**A**) miR-126-3p, (**B**) miR-26a, and (**C**) miR-133a showed increased expression in serum from TTC patients, compared to healthy donor and TTC-recovery, respectively (*n* = 3). (**D**) Epi had a higher expression in serum from TTC patients than in healthy donor and TTC recovery (*n* = 3). Results are presented as means ± SD. Scatter plots show the value of every experiment. *P < 0.05, **P < 0.01, ***P < 0.001, ****P < 0.0001 determined by one-way ANOVA with Holm-Sidak post-hoc test

Finally, to connect the main findings in this study with clinical practice, the serum levels of miR-126-3p and Epi were measured using blood samples from TTC patients at acute and reversed phases. Three TTC patients at the acute phase and three patients at the reversed phase as well as three healthy donors were recruited for the study. The results showed that serum levels of miR-126-3p, miR-26a, and miR-133a as well as Epi in TTC patients at the acute phase were significantly increased compared to serum from healthy donors (see Fig. 9A-D). Interestingly, TTC patients at the recovered phase showed a significant reversion of miRNAs and Epi (Fig. 9), supporting the roles of miR-126-3p and Epi for TTC pathogenesis.

## Discussion

The present study provides novel insight into the role of endothelial cell-derived exosomes in TTC pathogenesis, particularly focusing on their involvement in catecholamine-induced cardiac dysfunction and arrhythmogenesis. Our findings underscore the potential significance of exosomal miRNAs, particularly miR-126-3p, in mediating these effects, suggesting potential diagnostic and therapeutic targets for TTC management.

The exosomes secreted by hiPSC-ECs maintained intracellular Ca^2+^ homeostasis and promoted cardiomyocyte survival [14]. Endothelial cells release cardioprotective exosomes, potentially contributing to ischemic preconditioning [30, 31]. This suggests the intriguing possibility of endothelial communication with neighboring cardiomyocytes via exosomes. Our study successfully isolated exosomes from HCMECs, confirmed by the presence of well-known exosome markers and the absence of intracellular contaminants. This meticulous characterization ensures the purity and integrity of our exosome preparations, reinforcing the reliability of subsequent experimental outcomes. The observed uptake of endothelial exosomes by hiPSC-CMs underscores their potential role in mediating cellular communication and influencing cardiomyocyte function. This uptake mechanism establishes a direct link between endothelial cells and cardiomyocytes, implicating exosomes as key mediators of intercellular signaling.

The exosomes secreted by hiPSC-derived cardiac cells can improve myocardial recovery without increasing the frequency of arrhythmia-related complications [32]. The administration of hiPSC-EC exosomes can improve myocardial contractile function and alleviate adverse left ventricular remodeling post myocardial infarction (MI), without increasing the frequency of arrhythmias [14]. A previous study has indicated that extracellular vesicles (EVs) secreted by cardiosphere-derived cells suppressed arrhythmogenesis in arrhythmogenic cardiomyopathy (ACM) [33]. The response of cells to exosome treatment can be influenced by various factors, including the specific characteristics of the exosomes, the recipient cell’s phenotype, and the cellular microenvironment. Endothelial cell-derived exosomes contain various bioactive substances such as miRNAs, proteins, and cytokines. These substances may modulate the electrophysiological properties of cardiac cells, including ion channel function and action potential characteristics, thereby influencing the stability of cardiac rhythm.

Paracrine signaling from human mesenchymal stem cells (MSCs) has the potential to regulate ion channel/pump activity in cardiomyocytes, improve excitation-contraction coupling, and mitigate cardiac fibrosis [34, 35]. Exosomes from ECs under lipopolysaccharide (LPS) stimulation (LPS-EC-Exo) enhanced cell viability and attenuated cardiomyocyte injury [36]. Exosomes derived from M2 macrophage (M2‑exos) reduced the protein expression and current density of KCa3.1, resulting in a longer APD in pacing HL‑1 cells [37]. However, no studies have investigated the direct effect of exosomes derived from endothelial cells on the electrophysiological properties of cardiomyocytes. Our investigations revealed that exosomes from Epi-challenged HCMECs mimicked the Epi-induced dysfunction in cardiac ion channels and triggered arrhythmias by influencing ion channel expression and activity. This suggests that endothelial cell-derived exosomes may contribute to the modulation of cardiac ion channel functions.

There is now direct evidence that microRNAs biophysically modulate the cardiac action potential by direct binding to ion channels, in addition to the canonical RNA interference (RNAi) mechanism that regulates gene expression post-transcriptionally [38, 39]. miR-133a-3p caused repolarization abnormalities and significantly increased I_Ca−L_ in atrial cardiomyocytes [40]. miR-214-3p prolonged APD and Ca^2+^ decay, while miR-140 and miR-208a shortened APD [41]. Overexpression of miR-1 in myocytes resulted in a significant increase in the amplitude of the inward Ca^2+^ current, flattening of Ca^2+^ transients voltage dependence, and an enhanced frequency of spontaneous Ca^2+^ sparks^39^. The downregulation of HCN4 is linked to the upregulation of miR-423-5p in the sinoatrial node (SAN) of athletes and rodent models, while knockdown of miR-423-5p effectively reversed training-induced bradycardia in mice [42]. miR1 can reduce the density of the I_f_ current by repressing HCN4 translation in neonatal rat ventricular cardiomyocytes [42]. Conditional-knockout ECs miR-126 (miR-126^EC−/−^) mice exhibit significantly decreased cardiac function and increased cardiomyocyte hypertrophy, fibrosis, and inflammatory factor expression [43]. Circulating miRNAs, including miR-1, miR-133a, miR-16, and miR-26a, can be utilized for diagnosing acute TTC and even distinguishing TTC from acute myocardial infarction (MI) [26, 44]. In our study, serum levels of miR-126-3p, miR-26a, and miR-133a as well as Epi in TTS patients at the acute phase were significantly increased compared to serum from healthy donor or TTC patients at the recovered phase, supporting the roles of miR-126-39 and Epi for TTC pathogenesis. Furthermore, the levels of miR-16, miR-26a, miR-133a, and miR-126-3p in exosomes derived from Epi-treated HCMECs were elevated compared to those in exosomes from non-Epi-challenged HCMECs. This suggests that these miRNAs may mediate the observed exosome effects and play a crucial role in the development of TTC. Exosomes derived from HCMECs overexpressing miR-126-3p (Exo-mimic) exhibited effects on APs, arrhythmic events, and ion channel expression, similar to those induced by Epi. These findings imply that miR-126-3p is involved in catecholamine-induced ion channel dysfunction and arrhythmias under conditions of catecholamine excess.

It has been reported that miR-133a regulates RGS3 in gastric cancer, and the miR-133a-RGS3 axis may be involved in the malignant progression of the disease [45]. Similarly, miR‑126‑3p has been observed to inhibit proliferation, migration, invasion, and angiogenesis in triple‑negative breast cancer cells by targeting RGS3 [46]. However, how miR-126-3p regulates the contractile function and electrophysiological properties of cardiomyocytes is poorly understood. Our study examined whether RGS3 is the target of miR-126-3p and how it is regulated by miR-126-3p in HCMECs and hiPSC-CMs by different validation experiments. The results show that miR-126-3p can suppress RGS3 expression by targeting the RGS3 gene, providing mechanistic insight into the role of miR-126-3p in modulating cardiac ion channel function and suggesting potential therapeutic targets for arrhythmia management. It is known that RGS proteins including RGS3 can negatively regulate the activity of G proteins including Gs, Gi, and Gq [47, 48]. Since G proteins are coupled to adrenoceptors, the inhibitory effect of miR-126-3p on RGS3 may mimic or enhance catecholamine effects that depend on stimulation adrenoceptor/G protein signaling by reducing RGS3 effect on G protein activity, as observed in the current study. Notably, Epi could increase miR-126-3p in HCMECs by stimulating G protein-coupled adrenoceptors, including α-, β-, and D1-receptors, which was observed in the current study, and high-concentration catecholamine could cause ion channel dysfunction in cardiomyocytes via stimulating those three types of receptors, which was detected in our previous studies [24, 49, 50]. Taken together, it can be assumed that in the setting of high-level catecholamine, miR-126-3p in endothelial cells can be upregulated and released through exosomes to cardiomyocytes, and in turn enhance catecholamine effects in cardiomyocytes by increasing effects of G protein-related signaling. These findings underscore the intricate regulatory networks underlying endothelial exosome-mediated modulation of cardiac function and highlight the potential therapeutic relevance of targeting these pathways in cardiac arrhythmias.

Undoubtedly, there are some limitations in the present study. The study predominantly relies on in vitro models utilizing HCMECs and hiPSC-CMs. While these models provide valuable insights into cellular interactions and mechanisms, they may not fully recapitulate the complex in vivo cardiac microenvironment. TTC patient-specific hiPSC clone not included for disease phenotype. The study primarily focuses on the role of miR-126-3p carried by endothelial exosomes. Other miRNAs and non-coding RNAs present in exosomes may also contribute to the observed effects. Exploring the broader miRNA profile and potential interactions between different miRNAs could provide a more comprehensive understanding of exosome-mediated regulation. The direct connection between catecholamine-induced arrhythmias and TTC remains unclear. Although the study provides compelling in vitro evidence, further validation in animal models or clinical studies is necessary to confirm the relevance and translational potential of the findings.

Our findings suggest that endothelial exosomes, particularly miR-126-3p, contribute to catecholamine effects on modulating cardiac electrophysiology and arrhythmogenesis, which may provide valuable information about the pathogenesis of catecholamine-induced cardiac dysfunction and novel therapeutic strategies for managing arrhythmias associated with TTC.

## Electronic supplementary material

Below is the link to the electronic supplementary material.

Supplementary Material 1

Supplementary Material 2

Supplementary Material 3

## Data Availability

All data generated and/or analyzed during this study are available from the corresponding author upon reasonable request.
