# Supplementary figures and images for: Exosomal mir-126-3p derived from endothelial cells induces ion channel dysfunction by targeting RGS3 signaling in cardiomyocytes: a novel mechanism in Takotsubo cardiomyopathy
Source: Stem Cell Res Ther. 2025 Feb 4;16:36. doi: 10.1186/s13287-025-04157-0 (PMC11792229; doi:10.1186/s13287-025-04157-0)

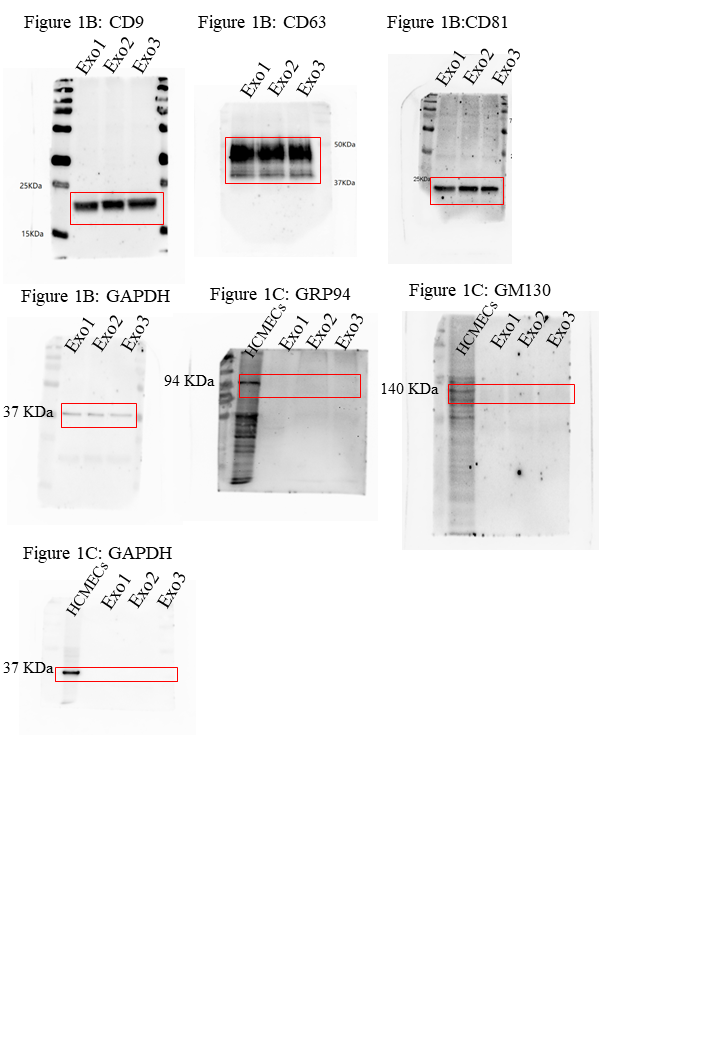

Supplement: Supplementary file 1 — Supplementary Material 1 [file 13287_2025_4157_MOESM1_ESM.tif]

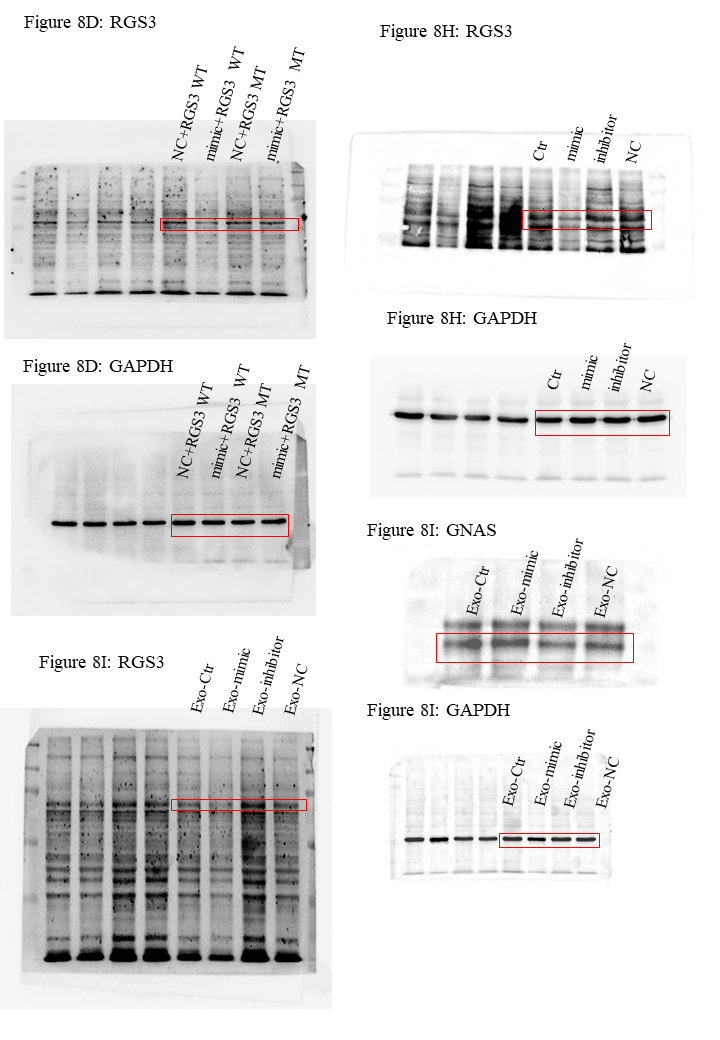

Supplement: Supplementary file 2 — Supplementary Material 2 [file 13287_2025_4157_MOESM2_ESM.tif]
