# Supplementary material for: Exosomal mir-126-3p derived from endothelial cells induces ion channel dysfunction by targeting RGS3 signaling in cardiomyocytes: a novel mechanism in Takotsubo cardiomyopathy
Source: Stem Cell Res Ther. 2025 Feb 4;16:36. doi: 10.1186/s13287-025-04157-0 (PMC11792229; doi:10.1186/s13287-025-04157-0)
Supplement: Supplementary file 3 — Supplementary Material 3 [file 13287_2025_4157_MOESM3_ESM.docx]

**Exosomal miR-126-3p Derived from Endothelial Cells Induces Ion Channel Dysfunction by Targeting RGS3 Signaling in Cardiomyocytes: A Novel Mechanism in Takotsubo Cardiomyopathy**

Xuehui Fan^1*#^, GuoqiangYang^2*^, Yinuo Wang^3^, Haojie Shi^3^, Katja Nitschke^4^, Katherine Sattler^5^, Mohammad Abumayyaleh^5^, Lukas Cyganek^6^, Philipp Nuhn^4^, Thomas Worst^4^, Bin Liao^7^, Gergana Dobreva^3^, Ibrahim El-Battrawy^8^, Daniel Dürschmied^5^, Xiaobo Zhou^1#^ and Ibrahim Akin^5^

^1^Department of Cardiology, Angiology, Hemostaseology and Medical Intensive Care, Medical Faculty Mannheim, University Medical Centre Mannheim (UMM), Heidelberg University, Mannheim, Germany; Key Laboratory of Medical Electrophysiology of the Ministry of Education, Medical Electrophysiological Key Laboratory of Sichuan Province, Institute of Cardiovascular Research, Southwest Medical University, Luzhou, Sichuan, China; Department of Cardiology, The Affiliated Hospital of Southwest Medical University, Luzhou 646000, China; European Center for AngioScience (ECAS) and German Center for Cardiovascular Research (DZHK) partner site Heidelberg/Mannheim, Mannheim, Germany.

^2^Department of Cardiology, Angiology, Hemostaseology and Medical Intensive Care, Medical Faculty Mannheim, University Medical Centre Mannheim (UMM), Heidelberg University, Mannheim, Germany; Acupuncture and Rehabilitation Department, The Affiliated Traditional Chinese Medicine Hospital of Southwest Medical University, Luzhou, 646000, China.

^3^Department of Cardiovascular Genomics and Epigenomics, European Center for Angioscience (ECAS), Medical Faculty Mannheim, Heidelberg University, Mannheim, Germany

^4^Department of Urology and Urosurgery, Medical Faculty Mannheim, Heidelberg University, Mannheim, Germany.

^5^Department of Cardiology, Angiology, Hemostaseology and Medical Intensive Care, Medical Faculty Mannheim, University Medical Centre Mannheim (UMM), Heidelberg University, Mannheim, Germany; European Center for AngioScience (ECAS) and German Center for Cardiovascular Research (DZHK) partner site Heidelberg/Mannheim, Mannheim, Germany.

^6^Stem Cell Unit, Clinic for Cardiology and Pneumology, University Medical Center Göttingen, Göttingen, Germany; DZHK (German Center for Cardiovascular Research), Partner Site, Göttingen, Germany.

^7^Department of Cardiac Macrovascular Surgery, Affiliated Hospital of Southwest Medical University, 646000 Sichuan, China

^8^Department of Cardiology and Angiology, Bergmannsheil University Hospitals, Ruhr University of Bochum, 44789, Bochum, Germany; Institute of Physiology, Department of Cellular and Translational Physiology, Medical Faculty and Institut für Forschung und Lehre (IFL), Molecular and Experimental Cardiology, Ruhr University Bochum, Bochum, Germany.

* These authors contributed equally to this work.

^#^Corresponding author：

Xiaobo Zhou, MD.

First Department of Medicine, University Medical Centre Mannheim,

Theodor-Kutzer-Ufer 1-3, 68167 Mannheim, Germany.

Phone: 0049-621-383-1448. Fax: 0049-621-383-1474.

E-mail: xiaobo.zhou@medma.uni-heidelberg.de.

Xuehui Fan, Ph.D.

First Department of Medicine, University Medical Centre Mannheim,

Theodor-Kutzer-Ufer 1-3, 68167 Mannheim, Germany.

Phone: 0049-621-383-1448. Fax: 0049-621-383-1474.

E-mail: xiaobo.zhou@medma.uni-heidelberg.de.

**Table S1. Antibody list**

| **Antibody** | **Resource** | **Dilution** | **Cat#** |
| --- | --- | --- | --- |
| Anti-SSEA4 | ThermoFisher Scientific | 1:100 | A24866 |
| anti-OCT4 | ThermoFisher Scientific | 1:200 | A24867 |
| anti-SOX2 | ThermoFisher Scientific | 1:100 | A24759 |
| anti-TRA-1-60 | ThermoFisher Scientific | 1:100 | A24868 |
| Alexa Fluor™ 488 goat anti-mouse IgG3; for use with anti-SSEA4 | ThermoFisher Scientific | 1:250 | A24877 |
| Alexa Fluor™ 488 donkey anti-rat; for use with anti-SOX2 | ThermoFisher Scientific | 1:250 | A24876 |
| Alexa Fluor™ 594 donkey anti-rabbit; for use with anti-OCT4 | ThermoFisher Scientific | 1:250 | A24870 |
| Alexa Fluor™ 594 goat anti-mouse IgM; for use with anti-TRA-1-60 | ThermoFisher Scientific | 1:250 | A24872 |
| CD9 Polyclonal Antibody | ThermoFisher Scientific | 1:1000 | PA5-11559 |
| CD63 Monoclonal Antibody | ThermoFisher Scientific | 1:250 | 10628D |
| CD81 Monoclonal Antibody | ThermoFisher Scientific | 1:500 | MA5-13548 |
| GM130 Antibody (B-10) | Santa Cruz | 1:500 | sc-55591 |
| Anti-GRP94 antibody | Abcam | 1:1000 | ab13509 |
| Anti-Cardiac Troponin T antibody [EPR20266] | Abcam | 1:100 | ab209813 |
| Anti-α-Actinin antibody | Sigma | 1:100 | A7811 |
| Goat anti-Rabbit IgG (H+L) Highly Cross-Adsorbed Secondary Antibody, Alexa Fluor™ 568 | ThermoFisher Scientific | 1:100 | A-11036 |
| Goat anti-Mouse IgG (H+L) Highly Cross-Adsorbed Secondary Antibody, Alexa Fluor™ Plus 488 | ThermoFisher Scientific | 1:100 | A32723 |
| RGS3 Monoclonal antibody | Proteintech | 1:1000 | 66790-1-Ig |
| GAPDH Mouse Monoclonal Antibody [Clone ID: 6C5] | Origene | 1:2000 | 5G4-6C5 |
| GNAS Polyclonal antibody | Proteintech | 1:2000 | 10150-2-AP |

**Table S2. Primers for real-time polymerase chain reaction (qPCR)**

| **Gene name** | **Cat No. Primers** | **Company** |
| --- | --- | --- |
| CACNA1C (L-type Ca^2+^ channel) | PPH01378G | Qiagen |
| SCN5A (Na^+^ channel, Nav1.5) | PPH01671F | Qiagen |
| SCN10A (Na^+^ channel, Nav1.8) | PPH15064A | Qiagen |
| SLC8A1 (NCX1) | PPH12509B | Qiagen |
| KCND3 (I_to_, Kv4.3) | PPH06923A | Qiagen |
| KCNQ1 (I_Ks_, Kv7.1) | PPH01419A | Qiagen |
| KCNH2 (I_Kr_, Kv11.1) | PPH01660A | Qiagen |

**Table S3. Primer sequence for qPCR**

| **Gene name** | **Primer sequence (5’-3’)** | **Company** | |
| --- | --- | --- | --- |
| RGS3 wildtype | F: CCG CTC GAG GGG CCA CTG GAG TCG AGC TC R: AAG GAA AAA AGC GGC CGC AAG GGT CAA GAA CAA GAA AT | | Sigma Aldrich |
| RGS3 mutant | F: CAA CCT TAA CCC TCA GAC CAC ACA GT  R: TAA GGT TGT TCC CAA AGC CCC CGA GG | | Sigma Aldrich |
| RGS3 | F: GTT CTG GTT GGC TTG TGA GG R: CCA GGT AGA GGT CAG AAC GG | | Eurofins |


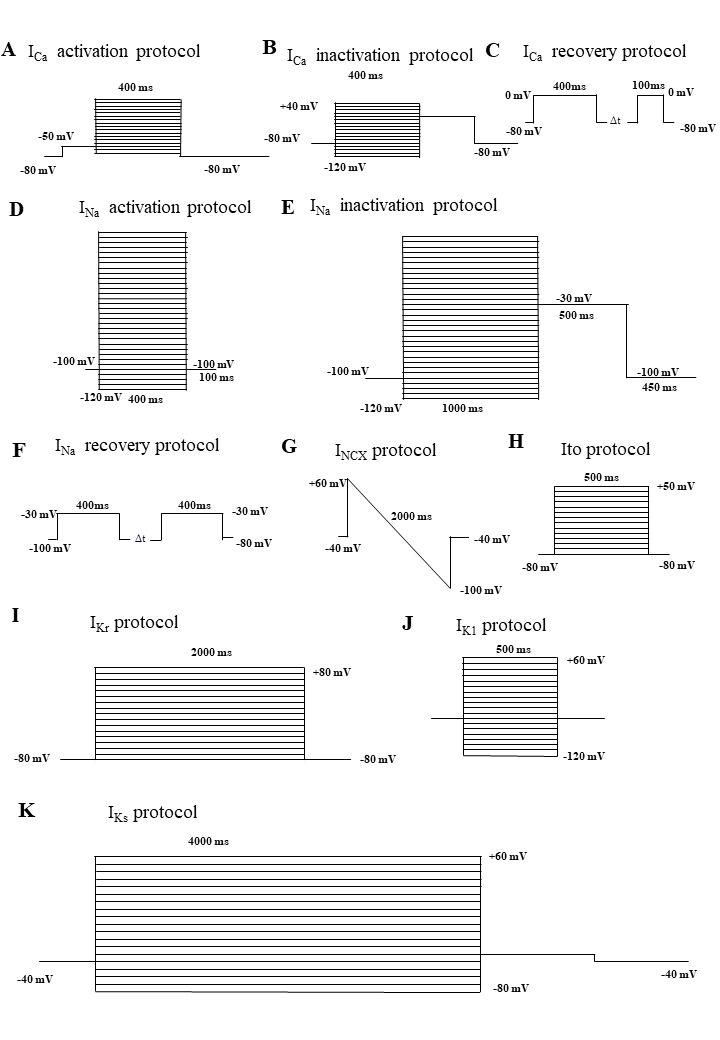


**Figure S1.** (A) Protocol for recording calcium current activation. (B) Protocol for recording calcium current inactivation. (C) Protocol for recording calcium current recovery. (D) Protocol for recording sodium current activation. (E) Protocol for recording sodium current inactivation. (F) Protocol for recording sodium current recovery. (G) Ramp protocol for recording NCX current. (H) Protocol for recording Ito. (I) Protocol for recording I_Kr_. (J) Protocol for recording I_K1_. (K) Protocol for recording I_Ks_.
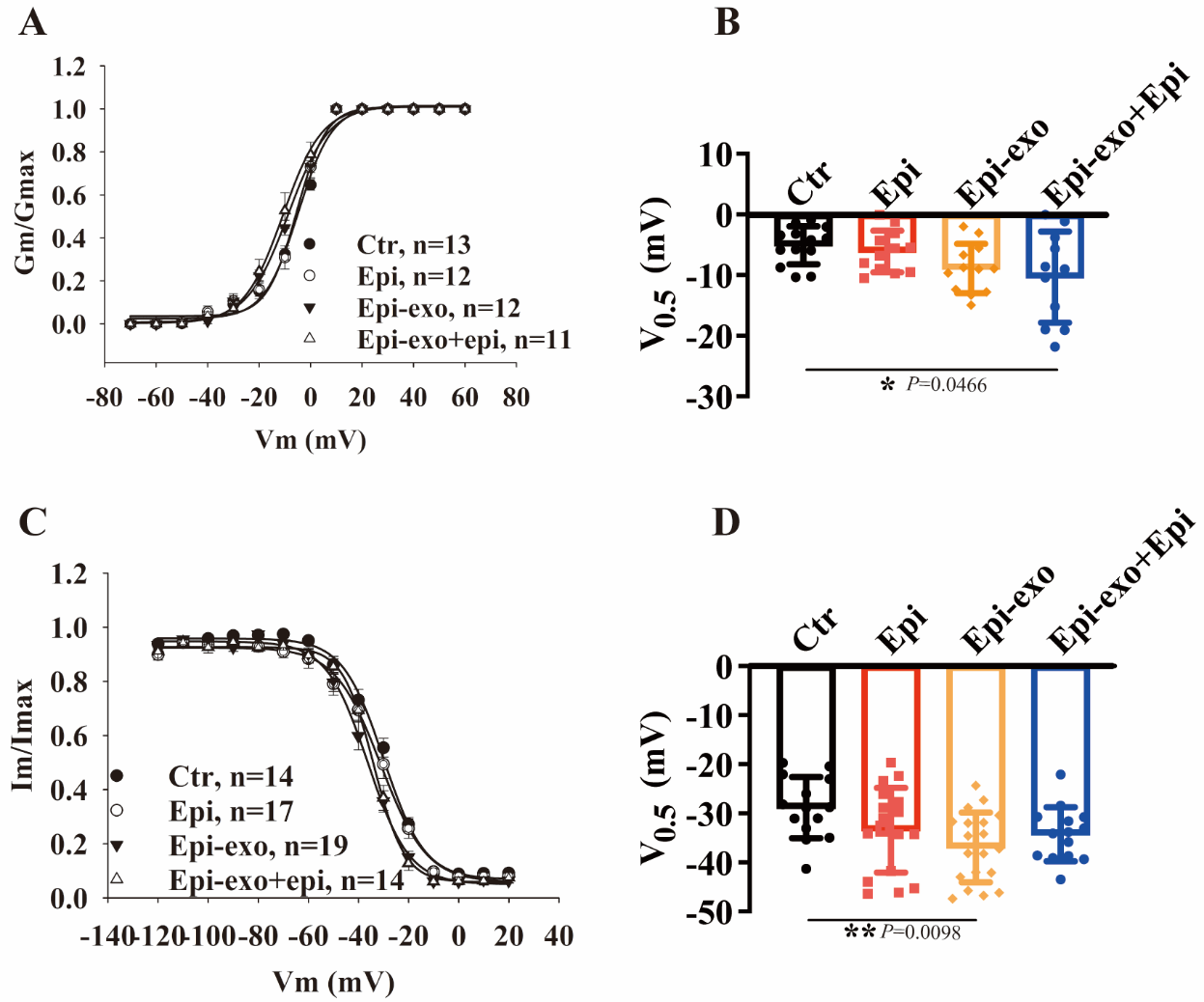


**Figure S2.** (A) Steady-state activation curves for I_Ca-L_ in indicated groups. (B) Mean values of potentials at 50% activation (V_0.5_) in indicated groups (n = 11-13 cells). (C) Voltage-dependent inactivation of I_Ca-L_ in cells of indicated groups. (D) Half-maximal inactivation potential (V_0.5_) in indicated groups (n = 14-19 cells). Results are presented as means ± SD. Scatter plots show the value of every measured cell. **P* < 0.05, ***P* < 0.01 determined by one-way ANOVA with Holm-Sidak post-hoc test.


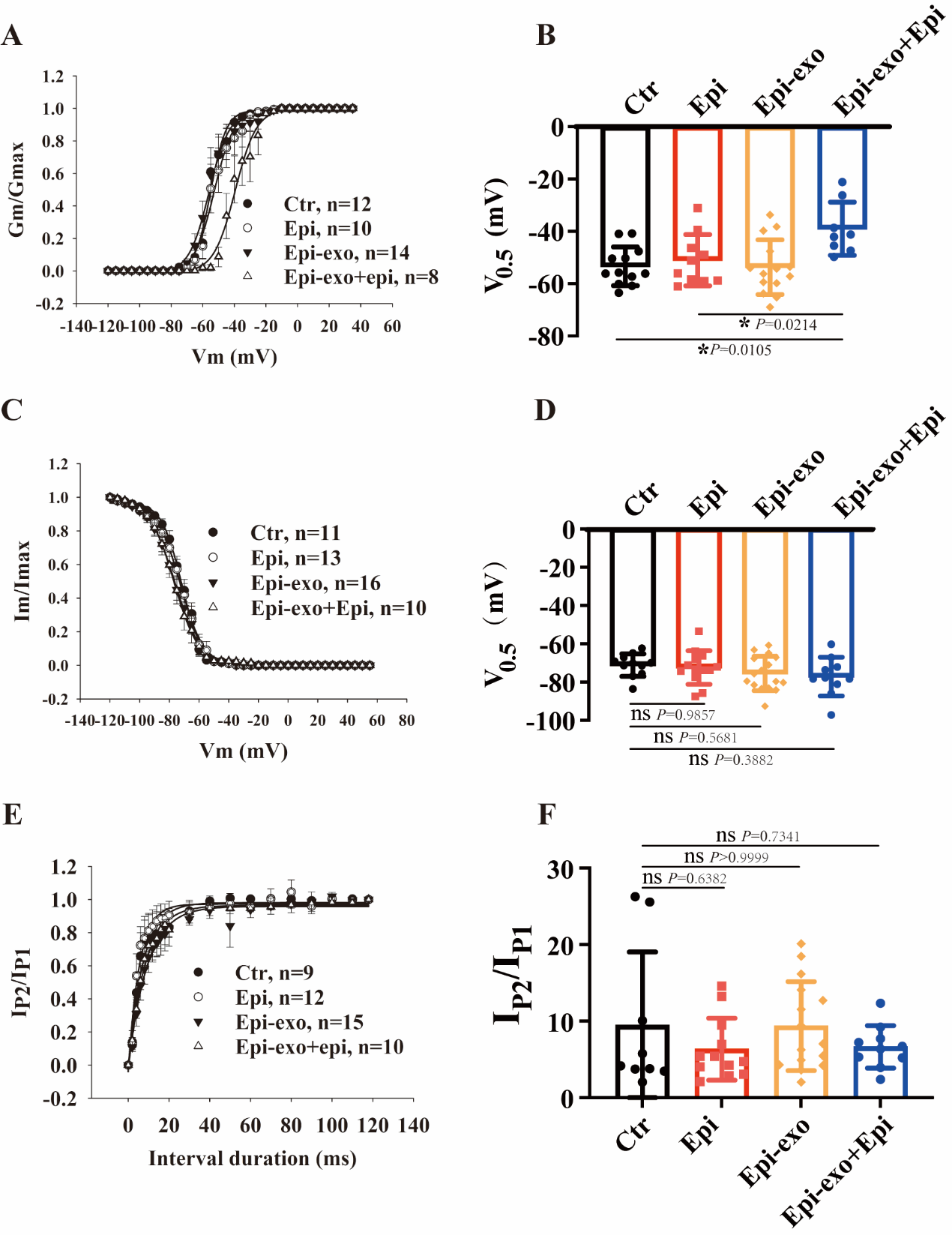


**Figure S3.** (A) Steady-state activation curves of peak INa in indicated groups. (B) Mean values of the half-maximal activation potential (V_0.5_) in indicated groups (n = 8-14 cells). (C) Voltage-dependent inactivation curves of peak INa in indicated groups. (D) Mean values of half-maximal inactivation potential (V_0.5_) in indicated groups (n = 10-16 cells). (E) Time course curves of recovery from inactivation in indicated groups. (F) Time constants (tau) of recovery from inactivation in indicated groups (n = 9-15 cells). Results are presented as means ± SD. Scatter plots show the value of every measured cell. **P* < 0.05 determined by one-way ANOVA with Holm-Sidak post-hoc test.


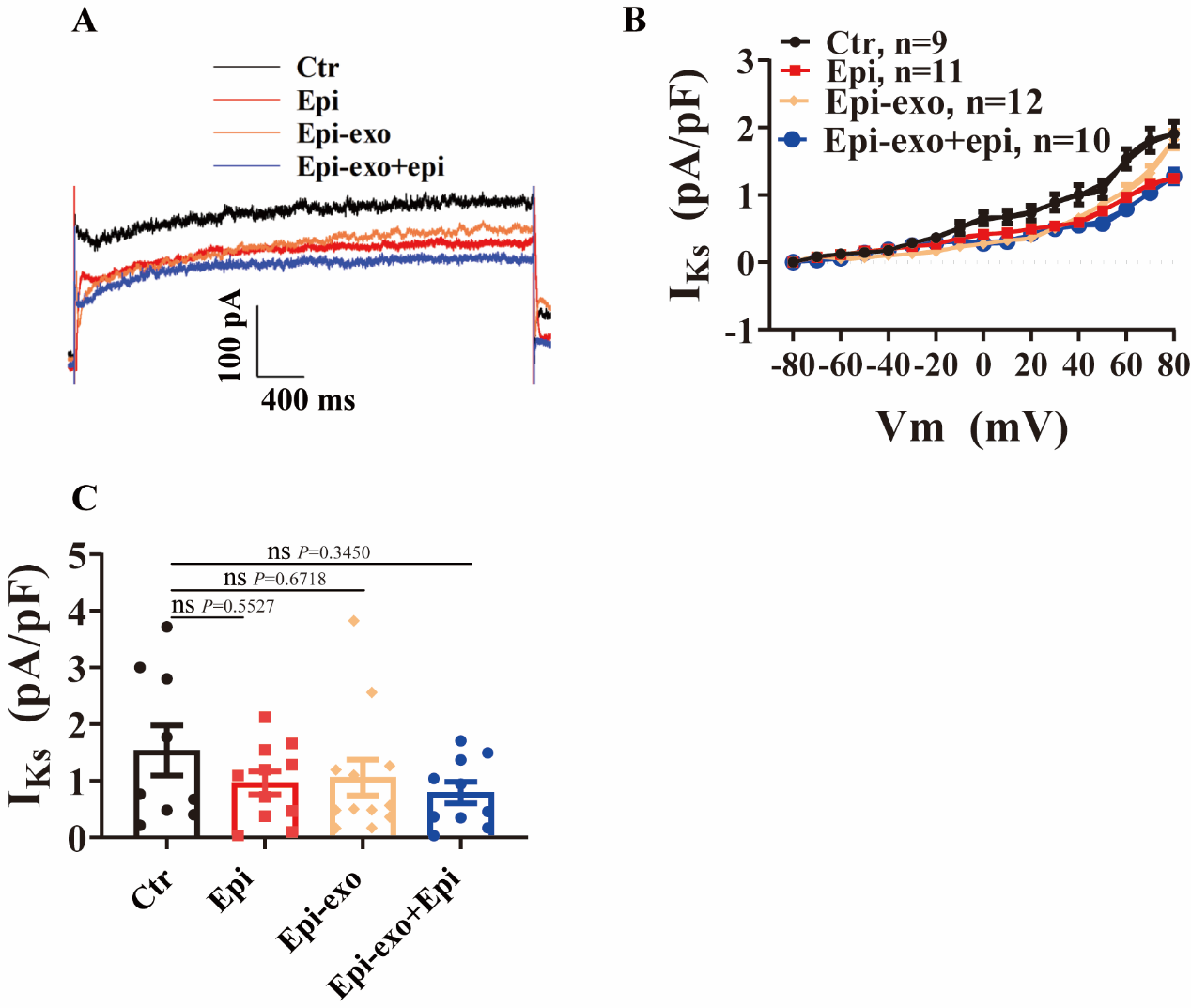


**Figure S4. Measurements of I_Ks_ in hiPSC-CMs treated with epinephrine or Epi-exo.** hiPSC-CMs were treated with either vehicle (Ctr) or 500 μM epinephrine (Epi), or exosomes secreted by HCMECs treated with Epi (Epi-exo), or Epi-exo plus Epi (Epi-exo+epi), respectively. Patch clamp whole cell recording techniques were used to measure slowly activating delayed rectifier potassium channel current (I_Ks_). (A) The raw traces of I_Ks_ at +60 mV in hiPSC-CMs in indicated groups. (B) I-V curves of I_Ks_ from -80 mV to +80 mV in hiPSC-CMs of indicated groups. (C) Current density of I_Ks_ at +60 mV in indicated groups (n = 9-12 cells). Data are shown as means ± SD. Numbers given in B represent the number of measured cells. Scatter plots (C) show the value of every measured cell. Results are presented as means ± SD. Scatter plots show the value of every measured cell.


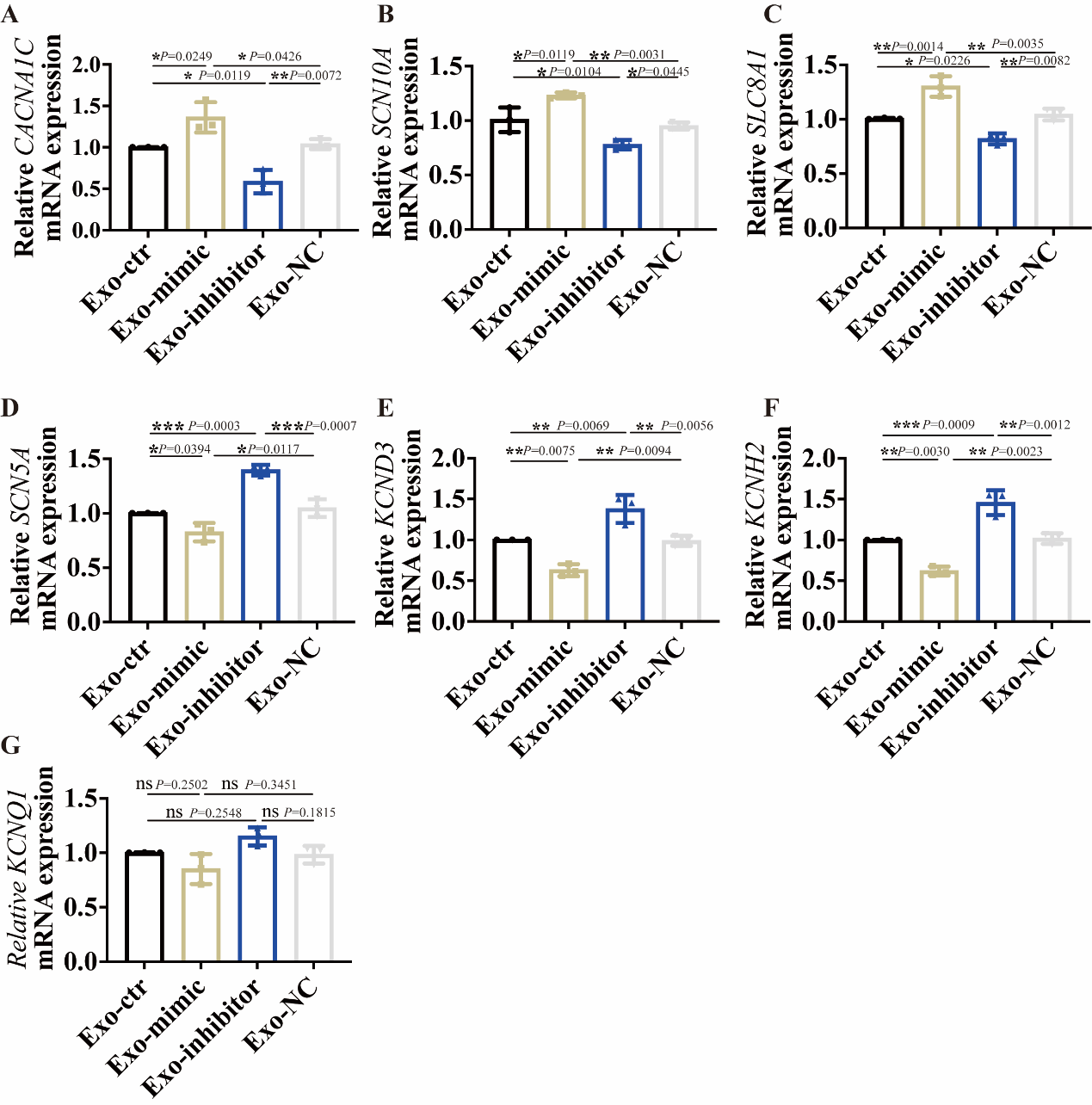


**Figure S5. Effects of exosomes derived from HCMECs transfected with miR-126-3p on ion channel expression in hiPSC-CMs.** hiPSC-CMs were treated with exosomes from HCMECs transfected with vehicle (Exo-ctr) or miR-126-3p mimic (Exo-mimic) or miR-126-3p inhibitor (Exo-inhibitor) or miR-126-3p inhibitor negative control (Exo-NC), respectively. qPCR was performed to measure gene expression. (A) The mRNA expression of *CACNA1C* in hiPSC-CMs of indicated groups (n = 3). (B) The mRNA expression of *SCN10A* in hiPSC-CMs of indicated groups (n = 3). (C) The mRNA expression of *SLC8A1* in hiPSC-CMs of indicated groups (n = 3). (D) The mRNA expression of *SCN5A* in hiPSC-CMs of indicated groups (n = 3). (E) The mRNA expression of *KCND3* in hiPSC-CMs of indicated groups (n = 3). (F) The mRNA expression of *KCNH2* in hiPSC-CMs of indicated groups (n = 3). (G) The mRNA expression of *KCNQ1* in hiPSC-CMs of indicated groups (n = 3). Results are presented as means ± SD. Scatter plots show the value of every experiment. **P* < 0.05, ***P* < 0.01, ****P* < 0.001 determined by one-way ANOVA with Holm-Sidak post-hoc test.
